# Forehead and In-Ear EEG Acquisition and Processing: Biomarker Analysis and Memory-Efficient Deep Learning Algorithm for Sleep Staging with Optimized Feature Dimensionality

**DOI:** 10.3390/s25196021

**Published:** 2025-10-01

**Authors:** Roberto De Fazio, Şule Esma Yalçınkaya, Ilaria Cascella, Carolina Del-Valle-Soto, Massimo De Vittorio, Paolo Visconti

**Affiliations:** 1 Department of Innovation Engineering, University of Salento, 73100 Lecce, Italy; suleesma.yalcinkaya@unisalento.it (Ş.E.Y.); ilaria.cascella@unisalento.it (I.C.); massimo.devittorio@iit.it (M.D.V.); paolo.visconti@unisalento.it (P.V.); 2 Facultad de Ingeniería, Universidad Panamericana, Aguascalientes 20296, Mexico; 3 Facultad de Ingeniería, Universidad Panamericana, Zapopan 45010, Mexico; cvalle@up.edu.mx; 4 Center for Biomolecular Nanotechnologies, Italian Technology Institute IIT, 73010 Arnesano, Italy; 5 Department of Health Technology, Technical University of Denmark, DK-2800 Lyngby, Denmark

**Keywords:** EEG acquisition, feature selection, sleep staging, wearable EEG, forehead EEG, in-ear EEG, sleep disorders, physiological signal analysis, two-step DL algorithm

## Abstract

Advancements in electroencephalography (EEG) technology and feature extraction methods have paved the way for wearable, non-invasive systems that enable continuous sleep monitoring outside clinical environments. This study presents the development and evaluation of an EEG-based acquisition system for sleep staging, which can be adapted for wearable applications. The system utilizes a custom experimental setup with the ADS1299EEG-FE-PDK evaluation board to acquire EEG signals from the forehead and in-ear regions under various conditions, including visual and auditory stimuli. Afterward, the acquired signals were processed to extract a wide range of features in time, frequency, and non-linear domains, selected based on their physiological relevance to sleep stages and disorders. The feature set was reduced using the Minimum Redundancy Maximum Relevance (mRMR) algorithm and Principal Component Analysis (PCA), resulting in a compact and informative subset of principal components. Experiments were conducted on the Bitbrain Open Access Sleep (BOAS) dataset to validate the selected features and assess their robustness across subjects. The feature set extracted from a single EEG frontal derivation (F4-F3) was then used to train and test a two-step deep learning model that combines Long Short-Term Memory (LSTM) and dense layers for 5-class sleep stage classification, utilizing attention and augmentation mechanisms to mitigate the natural imbalance of the feature set. The results—overall accuracies of 93.5% and 94.7% using the reduced feature sets (94% and 98% cumulative explained variance, respectively) and 97.9% using the complete feature set—demonstrate the feasibility of obtaining a reliable classification using a single EEG derivation, mainly for unobtrusive, home-based sleep monitoring systems.

## 1. Introduction

Polysomnography (PSG) is the standard diagnostic method for assessing sleep and associated disorders, involving the simultaneous monitoring of multiple physiological signals, such as the EEG, electrocardiogram (ECG), electrooculogram (EOG), electromyogram (EMG), and blood oxygen saturation (SpO_2_). However, PSG has notable limitations: it is typically conducted in specialized sleep laboratories, which may be unfamiliar or uncomfortable for patients. This setting can disrupt normal sleep patterns, leading to data that may not accurately reflect the individual’s typical sleep behavior [1,2].

Furthermore, this technique is particularly invasive, as it requires the placement of numerous sensors on the body, which can be uncomfortable, limit mobility, and disrupt natural sleep patterns. Moreover, PSG data interpretation is typically performed manually by trained specialists, introducing variability due to differences in experience and expertise among them. These issues highlight a fundamental problem: despite being the clinical gold standard, PSG is costly, invasive, restricted to laboratory settings, and has limited real-world validity, making it unsuitable for long-term and naturalistic sleep monitoring. As a result, a clear gap exists between the high-quality but impractical data obtained in clinical laboratories and the need for more accessible, comfortable, and reliable solutions that capture habitual sleep patterns. To overcome these limitations, wearable and portable sleep monitoring devices for home use and everyday environments have been developed. These systems are designed to minimize user discomfort, eliminate the need for specialized facilities, and enable long-term sleep tracking over multiple nights, unlike the typically single-night PSG assessments [2,3]. In particular, home-based monitoring applications would allow individuals to record their sleep in familiar settings, yielding data that more accurately reflects their habitual patterns. Similarly, wearable devices provide unobtrusive, continuous monitoring that can be integrated into daily life. Certain environments, such as space missions, present unique challenges where sleep monitoring is both critical and difficult. Astronauts’ sleep can be significantly disrupted by factors such as microgravity (requiring fixed sleeping positions), psychological stress, continuous background noise from onboard systems, prolonged exposure to artificial lighting, and the absence of natural light–dark cycles, which affect circadian regulation [4,5]. These factors make sleep monitoring crucial for astronauts, as poor sleep quality can impair cognitive function, reaction times, and overall mission performance. Thus, there is a clear demand for continuous, non-invasive, and portable sleep monitoring solutions in such resource-constrained environments [6,7].

To address these challenges, the SOMNIIA MONITOR project—funded by the Italian Space Agency and serving as the framework of this study—proposes a novel wearable polysomnograph in the form of a sleep mask. This device represents a significant advancement, integrating multiple biosensors into an ergonomic and comfortable design for reliable and non-invasive sleep monitoring [8]. Specifically, the mask incorporates ultra-thin, flexible aluminum nitride (AlN) piezoelectric sensors for tracking eye movements and monitoring heart and respiratory rates. It also features EEG and ECG acquisition sections equipped with dry electrodes, eliminating the need for conductive gels, as well as photoplethysmography (PPG) sensors for measuring SpO_2_ and heart rate variability. Inertial sensors are included to monitor body posture and movement, aiding in artifact reduction, while integrated temperature sensors record body temperature throughout the sleep cycle. The onboard microcontroller handles signal acquisition, conditioning, processing, and wireless transmission to a local host, where machine learning (ML) and deep learning (DL) algorithms perform automated sleep staging and real-time evaluation. The project aims to support and monitor the well-being and performance of astronauts during missions. Among the various bio-signals recorded during PSG, EEG remains the most informative modality for classifying sleep stages, diagnosing sleep disorders, and evaluating overall sleep quality [9]. The SOMNIIA MONITOR sleep mask would enable the acquisition of EEG signals through non-invasive sensors and other bio-signals, allowing for automated sleep staging through ML/DL algorithms. The proposed research aims to support the development of the sleep mask and the related software application for the SOMNIIA MONITOR project. In particular, the paper introduces a custom-designed experimental setup for capturing EEG signals from both the forehead and in-ear regions, aiming to facilitate robust sleep monitoring [10]. Then, a memory-efficient DL sleep staging algorithm was proposed to classify sleep from a low-dimensional feature set extracted from a single frontal derivation.

While sleep staging has traditionally relied on manual scoring of polysomnographic data by expert clinicians, recent advances have increasingly shifted toward automated approaches using machine learning techniques. The success of these methods, however, hinges on identifying features that are both meaningful and physiologically significant. The inclusion of irrelevant or redundant features can increase computational complexity, introduce noise, and ultimately reduce model accuracy and agreement with expert scoring [11]. Therefore, targeted feature selection is crucial for enhancing classification accuracy, reducing computational burden, and enabling reliable real-world applications [10].

The primary aspect of this study is the selection of physiologically meaningful EEG features relevant to sleep staging and the detection of sleep disorders. EEG signals exhibit variations in temporal, spectral, and nonlinear characteristics across sleep stages; therefore, combining features from these domains, it is possible to obtain a more comprehensive representation of brain activity. Time-domain features such as standard deviation, skewness, kurtosis, and zero-crossing rate capture amplitude fluctuations and waveform complexity, reflecting transitions between sleep stages [12]. Frequency-domain features, including delta, theta, alpha, and beta power, along with spectral slope and spectral edge frequency, reveal stage-specific oscillatory patterns and are also sensitive to pathological conditions like sleep deprivation and insomnia [13]. Additionally, non-linear features such as Lempel–Ziv complexity and Rényi entropy offer insight into brain dynamics and have demonstrated relevance in identifying stress-related or disordered sleep [14,15].

The main contributions of the proposed work are:

- An in-depth review of wearable EEG solutions utilizing forehead and in-ear placements, emphasizing their effectiveness in accurate and unobtrusive sleep stage classification and disorder detection.
- The development of a custom experimental setup for reliable EEG signal acquisition from both the forehead and in-ear regions.
- A feature set was identified based on an extensive literature review that supported their physiological relevance, followed by the application of mRMR and PCA to reduce its dimensionality using data from an open-access EEG database.
- A MATLAB-based (version 24.1) feature extraction tool was developed to process forehead EEG signals collected under various experimental conditions, enabling the analysis of correlations and trends related to the subject’s physiological state.
- A two-step ensemble classification approach was implemented, based on LSTM-based models trained to enable 5-class sleep staging.

The remainder of this article is structured as follows: Section 2 provides a review of the scientific literature on systems and algorithms for EEG acquisition for sleep monitoring, using a reduced number of forehead, ear, and in-ear derivations. Section 3 presents the experimental setups used for EEG acquisition, as well as the feature selection, extraction, and analysis aimed at sleep staging and the detection of sleep disorders. Section 4 includes signal acquisition tests from the forehead and in-ear regions, performance evaluations of the signal processing pipeline, and the training and testing of the proposed two-step sleep stage classification model. Finally, Section 5 discusses the findings, interprets the significance of the extracted features, and evaluates the performance of the developed deep learning framework in the context of automated sleep analysis.

## 2. Literature Analysis

In recent years, the development of wearable devices for EEG acquisition has opened up new perspectives in sleep monitoring, enabling increasingly compact, non-invasive and home-compatible solutions. Among these, particular attention has been given to systems using single or reduced numbers of forehead and ear/in-ear derivations; these last configurations offer advantages in terms of practicality, comfort, and signal quality. The following sections examine the main studies in the literature on devices and algorithms for forehead and in-ear/ear EEG acquisition. The literature review focuses on the architectural aspects and validation of acquisition systems, with particular emphasis on device setup, electrode configuration, and integration with advanced algorithms for automated sleep staging and feature extraction.

### 2.1. Overview of Forehead EEG Acquisition Systems and Algorithms for Sleep Monitoring

Several wearable systems have been proposed for non-invasive acquisition of forehead EEG. One example is Haru Sleep [16], a lightweight patch-style device with silver-based electrodes and Bluetooth transmission to a tablet. The model was tested with different electrode configurations to evaluate its performance, considering 1–6 channels. The best results for the Haru Sleep dataset were achieved when all channels were included. It integrates signal acquisition, preprocessing, and a DL-based sleep staging algorithm, reaching 78.6% accuracy and 73.4% F1-score, comparable to PSG. Similarly, the CGX patch [17], with electrodes at Fp1, Fp2, and AFz, with the latter as the reference. Three derivations—Fp1-AFz, Fp2-Fp1, and Fp2-AFz—are extracted from the three signals and compared with EEG signals from 32 channels, achieving 89% accuracy for REM epochs using spectral scoring, which confirms its reliability as a cost-effective alternative.

In addition to patch-type devices attached to the forehead, the Dreem Headband [18] employs dry EEG electrodes positioned at O1, O2, FpZ, F7, and F8 and a DL algorithm operating on EEG signals acquired by Ag-AgCl cup electrodes were also placed on the scalp to compare the result with PSG, achieving 83.5% ± 6.4% accuracy across five sleep stages, closely matching PSG results. Textile-based systems further improve comfort and wearability. For instance, a flexible and stretchable headband with printed electrodes and embedded circuits [19] features twenty-four channels positioned at AF8, AF10, FP10, FP2, FP1, FP9, AF7, and AF9, enabling comprehensive signal acquisition. It enabled low-noise acquisition and 24 h monitoring, while another multi-modal headband combined six EEG sensors with an IMU and temperature sensing [20], achieving a high correlation with PSG signals (Pearson correlation coefficient = 0.94) and a minimal difference of 1 ms in RR intervals between IMU-derived and PSG ECG-derived measurements.

Recent studies have increasingly focused on integrating ML/DL methods to automate further and improve sleep staging from forehead EEG. In ref. [21], a forehead system with three dry electrodes (Fh1, Fh2 and Fhz forehead EEG channels) used wavelet-based feature extraction and ML models (light gradient boosting machine, random forest (RF), and support vector machine (SVM)) to classify sleep stages into awake, light sleep (LS), deep sleep (DS), and REM, achieving 90.25% accuracy when using a combination of Fhz and Fh-EOG.

Another study [22] used a single flexible Ag/AgCl electrode EEG channel (F4-M1) with a neural network (NN) architecture, combining convolutional (CNN) and recurrent neural networks (RNN), which was trained and tested using EEG data recorded from subjects with suspected sleep apnea or bruxism. CNN–RNN architecture, reporting 79.7% accuracy for five classes (W, N1, N2, N3, R) and up to 89.1% when reduced to three stages (W, Non-Rapid Eye Movement (NREM), R).

Finally, a comparative analysis of the previously discussed scientific works is presented in Table 1, where the proposed systems are compared on common aspects, including the used leads, electrode typology, acquired channel number, main functionality, and battery life. In this way, the strengths and limitations of the introduced scientific works can be brought out. Systems such as Haru Sleep [16] and Dreem Headband [18] offer convenient, non-invasive, and practical solutions for DL-based algorithms in sleep staging, achieving notable accuracies of 78.6% with a custom dataset provided from 30 subjects and 83.5% ± 6.4% with a custom dataset provided from 25 subjects, respectively, and both used a PSG device simultaneously and compared the obtained scoring accuracy. However, the accuracy of wearable devices remains slightly lower than that of clinical PSG systems. While dry electrode-based systems improve portability, they often suffer from increased signal noise. Forehead EEG devices typically rely on electrode positions such as Fp1, Fp2, Fpz, AFz, or custom forehead sites (e.g., Fh1, Fh2, Fhz), which are advantageous due to the absence of hair and ease of attachment. These placements enable stable overnight acquisition without requiring extensive preparation, while still capturing frontal slow-wave and spindle activity relevant for NREM staging. Multi-channel forehead configurations (e.g., Fp1-AFz, Fp2-Fp1, Fp2-AFz) have demonstrated improved robustness compared to single-lead setups [17]. Although accuracy remains slightly lower than that of full PSG, studies show that frontal derivations alone can achieve greater than 80% agreement with expert scoring, making them suitable for portable and home-based monitoring [18]. However, their anterior location reduces sensitivity to occipital alpha rhythms and REM-related ocular activity, representing a key trade-off between simplicity and signal diversity. Notably, the EEG sensors utilize flexible claw-shaped dry electrodes that effectively penetrate the hair to ensure consistent scalp contact, thereby enhancing signal quality. The use of stretchable printed electrodes and flexible dry electrodes presents promising advancements in comfort and signal acquisition; however, limited validation on small subject cohorts, combined with the lack of large-scale clinical trials and standardized algorithm benchmarking, as most models are tested on custom datasets [19,20]. These systems demonstrate significant progress toward accessible and efficient sleep monitoring, striking a balance between usability, cost, and accuracy.

### 2.2. Overview of Ear and In-Ear EEG Acquisition Systems and Algorithms for Sleep Monitoring

Many studies have focused on non-invasive EEG acquisition using in-ear devices, such as those presented in ref. [23] where the authors compared an in-ear EEG setup based on IDUN Technology’s Guardian Development Kit (250 Hz) [24] to PSG (SOMNOscreen Plus, 256 Hz) [25] in 10 subjects. PSG electrodes included six bipolar EEG leads, six unipolar leads with M1-M2 reference, and six leads for EOG. The analysis included two phases: first, temporal and frequency features were extracted from 30 s EEG windows, and hypnograms were plotted; then, the hypnograms were compared using the Jensen-Shannon Divergence Feature-based Similarity Index (JSD-FSI). The study revealed a strong similarity between in-ear and PSG EEG signals (JSD-FSI values: wakefulness, 0.61 ± 0.06; NREM, 0.60 ± 0.07; REM, 0.51 ± 0.08), with lower similarity in REM sleep, emphasizing the need for EOG data. These results support the reliability of in-ear EEG, while acknowledging limitations in detecting REM sleep.

Research on ear-based technologies, particularly with electrodes outside the auditory canal, is ongoing. On the other hand, flexible conductive fabrics represent a step forward in integrating electronic materials into wearable devices. In ref. [26], the authors developed an in-ear memory foam sensor. They conducted a study to assess the similarity between EEG signal characteristics recorded from the scalp and those obtained through their solution. The experimental setup consisted of a tubular device with two flexible, conductive fabric electrodes placed opposite each other. The two systems were compared in terms of sensitivity, specificity, and accuracy in distinguishing N2/N3 sleep stages from N1/W and NREM sleep from wakefulness. Cohen’s Kappa values were k = 0.65 and k = 0.60, showing substantial agreement. Similarly, in ref. [27], the authors presented an EEG earpiece made of memory foam and conductive fabrics for acquisition outside the ear canal. A comparison with a frontal setup evaluated impedance, signal quality, and alpha wave analysis. The study involved 10 participants with varying auricular sizes. The frontal setup had electrodes at Fp1 and Fp2, with a bias electrode on the inion and a reference electrode positioned between the inion and the ear. Using the OpenBCI Cyton device [28], alpha waves were observed with “closed eyes” but disappeared when the eyes were opened. In-ear recordings had over 98% validity, whereas the frontal setup had lower validity (46.36–78.67%) and more artifacts, particularly during the “open-eyes” condition. Acoustic stimulation synchronized with slow EEG oscillations was noted to improve sleep quality.

Building on the practical applications of in-ear EEG devices, in ref. [29], the authors presented a sleep monitoring system using generic earphones with an in-ear EEG setup. The earphones featured a tulip-shaped ear canal section, a tail for stability, and a main body to reduce movement artifacts. Two electrodes in each earphone captured EEG signals, with one as a reference and separate ground electrodes. The system utilized a four-channel ASIC (Application-Specific Integrated Circuit) amplifier (250 Hz, 14-bit) for signal recording. The study involved 10 participants who recorded EEG for 12 nights, divided into two groups, using a partial PSG setup and only the in-ear system. EEG data were preprocessed, and a sleep staging algorithm (RF) was trained on three datasets.

A recent study in [30] introduced the Lightweight In-ear Biosignal Sensing System (LIBS), a compact and cost-effective wearable device designed to capture EEG, EOG, and EMG signals from inside the ear canal. The system leverages a novel in-ear sensor made of flexible, highly conductive memory foam to ensure stable signal acquisition with minimal discomfort. By employing a non-negative matrix factorization (NMF) algorithm, the system successfully separates mixed biosignals into distinct EEG, EOG, and EMG components, preserving their integrity for sleep stage classification. The study demonstrated that LIBS achieves an impressive 95% accuracy in identifying sleep stages (N1, N2, N3, and REM), making it a viable alternative to traditional PSG for sleep monitoring.

Finally, Table 2 compares the previously analyzed scientific papers concerning systems for in-ear EEG acquisition and processing from the perspective of position, type, number of electrodes, device objective, and used algorithms.

In-ear systems typically record one or two channels with a reference on the mastoid, earlobe, or behind the auricle, enabling the acquisition of cortical activity with high similarity to scalp EEG [26,27]. The ear and in-ear EEG acquisition systems offer a promising solution for implementing discrete and effective sleep staging, allowing for high-quality and artifact-free signals compared to those acquired from the forehead, which is fundamental for reliable sleep classification.

The Guardian Development Kit exhibits strong signal similarity with PSG but faces challenges in REM detection due to the absence of EOG data [23]. Flexible conductive fabric electrodes, such as those used by Looney et al. [26] and Mandekar et al. [27], improve comfort and stability while maintaining reliable signal quality. With integrated EEG, EOG, and EMG acquisition and advanced signal separation, the LIBS system achieves a high sleep stage classification accuracy (95%), positioning it as a promising alternative for home-based sleep monitoring [30]. Overall, while each system has trade-offs between comfort, accuracy, and signal processing techniques, these advancements collectively enhance the feasibility of in-ear EEG for real-world applications.

## 3. Materials and Methods

This section presents the experimental setups used for EEG acquisition, as well as the feature selection, extraction, and analysis aimed at sleep staging and the detection of sleep disorders. This study utilizes two distinct data sources: a custom experimental setup (Section 3.1) for initial feature validation and physiological correlation, and the public BOAS dataset (Section 3.2) for the robust training and evaluation of our deep learning model.

### 3.1. Experimental Setups and Methodologies for EEG Acquisition

An innovative solution for biopotential acquisition from Texas Instruments (Dallas, TX, USA) is represented by the ADS1299 IC (Integrated Circuit) [31], along with its variants ADS1299-4 and ADS1299-6, which constitute a front-end solution for biopotential acquisition, such as EEG and ECG, characterized by lower amplitudes compared to other bioelectrical signals. These devices feature delta-sigma (ΔΣ) analog-to-digital converters (ADCs) with four, six, or eight channels, offering 24-bit resolution and low noise levels, enabling simultaneous sampling across all channels. Additionally, they integrate a Programmable Gain Amplifier (PGA) with user-configurable gain settings (1, 2, 4, 6, 8, 12, or 24 V/V). The IC includes a 4.5 V internal voltage reference and an internal oscillator that generates a 2.048 MHz clock signal. The front-end supports flexible electrode input configurations, including averaging for generating the patient’s bias signal, and offers lead-off detection for monitoring electrode disconnections.

The ADS1299 integrated front-end, thanks to the integrated PGAs, ensures lower noise and more precise gain matching between channels than a front-end realized with a discrete instrumentation amplifier. The ΔΣ architecture is renowned for its excellent noise performance. It works by oversampling the signal (sampling at a rate much higher than the Nyquist rate, e.g., the ADS1299 modulates at 2.048 MHz) and then using a digital filter to decimate the data to the output sampling rate (e.g., 250 Hz to 16 kHz). Summarizing, the ADS1299’s integrated design, which combines high-resolution ADCs, low-noise PGAs, and an active reference driver on a single chip, provides exceptional signal quality and noise suppression in a compact form factor ideal for wearable applications.

The ADS1299EEG-FE-PDK electronic board of Texas Instruments is an evaluation board for the ADS1299 IC. The kit includes the evaluation board and the modular motherboard EVM MMB0, which allows a computer to be connected via a USB port. The board offers various hardware configurations through jumpers, including bipolar and unipolar supply options, internal and external clock selection, and voltage reference choices. The provided software includes advanced analysis tools, such as a virtual oscilloscope, a histogram, a Fast Fourier Transform (FFT) analysis, and the ability to export raw EEG data for further processing. The ADS1299EEG-FE-PDK board is stacked on top of the EVM MMB0 motherboard via three headers (designated as JP2, JP3, and JP4) [31]. The front-end can be powered with three different voltage levels (+5 V, +3 V, and +1.8 V), supplied by the MMB0 host board via the J4 connector. Other voltages required for the operation of the EEG front-end are generated directly onboard through dedicated power management circuits. Additionally, the ADS1299 can be disabled by shorting the JP5 jumper. The ADS1299 features an integrated oscillator that generates a 2.048 MHz clock, with an accuracy that may vary by ±5% due to temperature variations. For greater precision, an external clock signal can be utilized. The device also includes an internal temperature sensor, whose output voltage can be converted into a temperature value using the following equation:

(1) $$Temperature\;\left({}^{\circ}C\right)=\left(\frac{Temperature\;Reading\;\left(\mu V\right)-145.300\;\mu V}{490\;\mu V/{}^{\circ}C}\right)+25\;{}^{\circ}C$$

The ADS1299EEG-FE-PDK enables the testing of internal and external clock configurations, allowing the use of either the integrated oscillator or an external clock source. Some output signals of the ADS1299 are accessible through the JP5 connector, which serves as a test point for signal measurement and analysis. The digital signals of the board, including those of the SPI interface, GPIO signals, and some control signals, are accessible through the JP3 connector.

**Figure 1:**
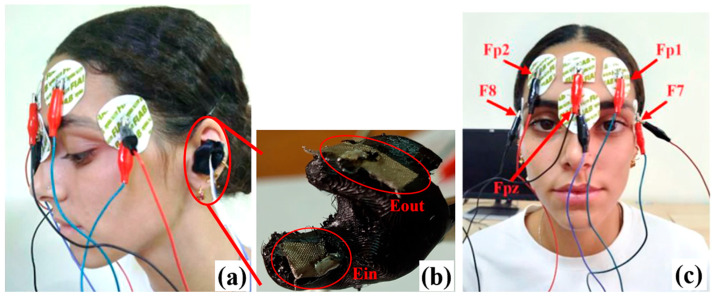
Electrode positioning for forehead and in-ear EEG acquisition: side view (**a**), developed earbud with two textile electrodes, called Ein and Eout (**b**), and front view with highlighted electrode positions according to the 10–20 International System (**c**). The uncropped images are available in the Supplementary Files.

In our experimental tests, the focus was initially directed toward the frontal region; specifically, the EEG signal was acquired by six electrodes positioned in the frontal areas of the cerebral cortex at locations Fp1, Fp2, F7, and F8, following the international 10–20 system for electrode placement (Figure 1a,c). The bias electrode was positioned in the upper part of the nose (nasion), ensuring a precise and stable recording. These areas are involved in cognitive and affective processes that vary throughout the sleep cycle, making them ideal for monitoring slow-wave EEG signals, which are essential for sleep monitoring. The disposable electrodes used feature a circular memory foam support and an Ag/AgCl sensor, contributing to the reduction in artifacts in the signal. The recorded signals include the following bipolar derivations: Fp2-Fp1, F8-F7, and F7-Fpz, representing the electrical potential difference between the two electrodes of each pair. The Fpz position, located at the center of the forehead between Fp1 and Fp2, was included in the analysis as it belongs to the sagittal line of the skull, which is useful for monitoring delta (0.5–4 Hz) and theta (4–7 Hz) waves, characteristic of drowsiness states, as well as beta waves (13–30 Hz), often associated with active cognitive processes and concentration.

For each subject, central and landmark electrode positions were determined using a flexible measuring tape (e.g., nasion–inion distance, head circumference). Based on these measurements, other positions were then determined. This systematic approach ensures that electrode placement is both accurate and reproducible across participants. In addition, documenting the raw measurements provides a valuable quality control metric, enabling verification of proper placement and allowing for retrospective checks of electrode localization in the event of anomalies during signal analysis. Such rigor is particularly important when comparing across subjects or sessions, where even small deviations in electrode position can influence the spatial resolution of spectral or topographical analyses.

Subsequently, an earbud was inserted into the left ear for the acquisition of in-ear EEG signals. The earbud, manufactured using 3D printing, was designed to fit perfectly into the ear’s anatomy. The earbud features two rectangular electrodes made of flexible, conductive fabric (Metal nylon), ensuring high flexibility and adaptability to the irregular surfaces of the ear canal (Figure 1b). Before the acquisition, conductive gel (Tecnocarta GECG260 Gel for ECG) was applied to both electrodes to optimize contact between the electrodes and the skin of the ear canal, reducing contact impedance.

**Figure 2:**
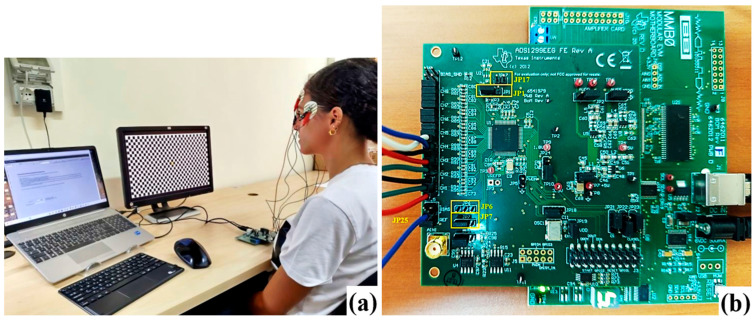
Experimental setup for forehead and in-ear acquisition (**a**) and evaluation board ADS1299EEG-FE-PDK with highlighted jumpers’ configuration (**b**). The uncropped images are available in the Supplementary Files.

The experimental setup used for EEG signal acquisition and processing includes the ADS1299EEG-FE-PDK electronic board, employed to amplify and acquire the EEG signal from the electrodes. The board configuration used for the measurements leveraged the Bias Drive functionalities integrated into the ADS1299 chip. This IC features a buffer that enables the voltage applied to the subject to be adjusted to minimize common-mode disturbances, such as power line interference. This voltage can be generated internally, at a midpoint between the chip’s positive (AVDD) and negative (AVSS) power supply, or provided externally. To use this signal, jumper JP1 was installed in positions 1–2, keeping the BIAS_SHD buffer input disabled by leaving JP17 jumper open. The board also features an external buffer with the same function, which was not used in the reported measurements; therefore, the JP6 jumper was not installed. Since the acquisition involved only bipolar derivations, a reference electrode was not employed. For this reason, jumper JP7 remained installed on the board, keeping the pin grounded through JP25, with the jumper placed between pins 5 and 6. Figure 2a shows the positions of the listed jumpers on the board. The EEG acquisition software features a graphical interface divided into four main sections. The “About” tab provides information on the firmware version loaded onto the board. The “ADC Register” tab enables the graphical configuration of control registers, allowing users to set acquisition board parameters. The “Analysis” tab offers tools for analyzing acquired data in both the time and frequency domains. Finally, the “Save” tab provides options for saving the acquired data.

In the first section of the “ADC Register” tab, the ADS1299 front-end can be configured by setting the output data rate to 1 kSPS. The integrated buffer for BIAS was also enabled, setting a polarization voltage equal to the midpoint between the positive (AVDD) and negative (AVSS) power supply. The necessary channels for acquisition were activated, while the remaining ones were disabled, configuring them in power-down mode. Each channel was set to acquire a bipolar derivation in normal electrode mode. In the LOFF and BIAS section, registers are available for lead-off detection, current control, and polarization regulation. In the lower section of the panel, called BIAS Control Registers, all channels were disabled since measuring the bias voltage applied to the subject was not required. Within the “Analysis” tab, three main tools are available: the Scope allows visualization of the acquired waveform signals, the Histogram represents the amplitude distribution of the harmonics, and the FFT enables frequency spectrum analysis of the recorded EEG signals. The signals collected by the board were stored in a 10 s time window, corresponding to 10,000 samples, and then saved in a text file (.txt). The data were subsequently preprocessed and plotted using a MATLAB (version 24.1) script. This script loads the acquired EEG data, removes the common mode, and applies a notch filter with a central frequency of 50 Hz to eliminate power line interference. The power spectrum of the signals was computed using the pspectrum() function to determine the power distribution across different bands and calculate power ratios. In practice, we confirmed the effectiveness of common-mode removal by visually inspecting raw EEG traces and power spectra across subjects. Residual artifacts—mainly transient muscle activity and occasional electrode displacement effects—were handled through preprocessing steps (bandpass filtering with 0.2 Hz and 44 Hz cut-off frequencies and 50 Hz notch filtering). Still, for the scope of this study, we relied on our preprocessing pipeline to ensure adequate signal quality for sleep staging. However, future work will focus on implementing algorithms, such as linear regression or Independent Components Analysis (ICA), to mitigate the eventual presence of artifacts resulting from eye movements, muscular contractions, and the electrocardiographic signal.

During the test, the contact impedance of electrodes was periodically checked using the Impedance Measurement Mode of the ADS1299. This built-in feature operates by injecting a known, physiologically benign alternating current (AC) of 6–24 nA at a specific frequency and measuring the resultant voltage drop across the electrode-skin interface to calculate impedance. By ensuring all electrodes maintain a low and stable impedance (typically below 10 kΩ), this method directly combats two major sources of signal degradation: it minimizes the attenuation of the microvolt-level EEG signals. It dramatically enhances the common-mode rejection capability of the differential amplifiers, which is essential for suppressing ubiquitous ambient electromagnetic noise.

The first tests to characterize the ADS1299 front-end focused on acquiring and processing EEG signals using the evaluation board, with exclusive consideration of the bipolar derivation Fp2-Fp1. Specifically, EEG signals were recorded under four different experimental conditions. In the first condition, the subject was at rest with their eyes open, without exposure to any stimulus. In the second condition, the subject remained at rest with their eyes closed, without any external stimuli. The third condition involved the recording of VEP, obtained through the presentation of a Pattern Reverse Visual Evoked Potential (PRVEP) [32], in which the subject observed a checkerboard pattern with alternating square colors at a frequency of 2 reversals per second (Figure 2b). Finally, in the fourth condition, the subject was exposed to an audio-visual stimulus by watching a video on a screen to analyze the brain’s response to this stimulation.

### 3.2. Selection and Analysis for Sleep Staging and Detecting Disorders of Sleep

Sleep staging, the classification of different phases (wakefulness, REM sleep, and NREM sleep), is a fundamental step in assessing sleep quality and detecting sleep-related disorders. The first step in accurate staging is the thorough analysis of physiological signals recorded during sleep, primarily the EEG, which enables the monitoring of variations in the subject’s brain activity.

A crucial aspect in ensuring staging accuracy is the selection of the most relevant features from the collected signals, as extracting the right features significantly impacts the performance of classification algorithms. Using an excessive number of features or those with low discriminative power increases computational complexity and introduces noise into the models, compromising the effectiveness of sleep staging. Therefore, a literature review has been conducted to examine recent and relevant scientific articles on EEG signal analysis for sleep staging and the identification of related disorders. This analysis identified EEG features in the time, frequency, and non-linear domains, highlighting how their combination represents the optimal choice for achieving satisfactory results for sleep analysis. The selected features are accompanied by their physiological significance, which helps to understand how they vary across different sleep stages and in relation to the subject’s specific conditions.

In the time domain, statistical features were calculated to capture the amplitude fluctuations and distribution characteristics of the EEG signal, which included maximum, minimum, mean, median, root mean square (RMS), and percentiles (25th, 50th, and 75th). Features such as variance, skewness, and kurtosis were particularly informative for identifying stage transitions and characterizing signal complexity. Variance and standard deviation increase from wakefulness through REM to N3, while the distributions of skewness and kurtosis become narrower across stages, with distinct peaks marking transitions [12]. Hjorth parameters were utilized to evaluate the activity and complexity of EEG signals, particularly within the sensorimotor and visual cortical regions. In the sensorimotor area, these parameters effectively distinguish wakefulness and REM sleep, characterized by higher activity levels, from NREM sleep, which exhibits lower activity. In the visual cortex, they help differentiate wakefulness from the REM stage based on higher signal activity and reduced complexity during wakefulness. Additionally, variations in Hjorth parameters can be used to detect unstable or fluctuating amplitude patterns within NREM sleep, which are indicative of diminished sleep quality [33]. Additional time-domain metrics included the Zero-Crossing Rate (ZCR), which decreases during NREM due to dominant slow-wave activity, and is reduced in posterior regions due to increased alpha and theta power replacing beta activity [34]. The Average Amplitude Change (AAC) and Clearance Factor were used to capture waveform complexity, which is typically higher during REM and wakefulness, and reduced during deep sleep [35]. The Interquartile Range (IQR) serves as a marker of signal irregularity, with higher values observed during wakefulness and light sleep, and lower values during REM and N3 sleep [36]. The Simple Square Integral (SSI) was also calculated to assess energy in sub-bands and declines from wakefulness to REM sleep, mirroring decreases in entropy and signal variability [37].

In the frequency domain, spectral features were computed to characterize the distribution of power across classical EEG bands. Total power was found to be higher during NREM than REM, and a reduction in delta, theta, and alpha power is a known indicator of sleep deprivation [38,39]. Relative Spectral Power (RSP) helps differentiate subjects with subjective versus objective insomnia, with increased alpha power (notably at Fp2) linked to stress-induced hyperarousal [40]. Additionally, Power ratios were analyzed across stages, revealing higher ratios during wakefulness and N1, with declines through N2 and N3, followed by a subsequent increase in the REM stage. The dominant frequency was used to track transitions across stages: alpha activity dominates in REM and N1 stages, whereas N2 and N3 show more variability with mixed-frequency components and lower delta power before arousals [13]. The slow wave index (SWI) provided insight into how alpha, theta, and delta bands relate to slow-wave activity. As the brain transitions from wakefulness to sleep, the alpha slow wave index (ASI) typically decreases due to a reduction in alpha band power, while the power in slower frequencies such as delta and theta increases [41,42].

The harmonic characteristics of EEG signals vary significantly across sleep stages; particularly, during wakefulness, the EEG exhibits a high center frequency of approximately 20 Hz with a relatively broad bandwidth of around 15 Hz, and the amplitude at the central frequency is relatively low [43]. In the N1 stage, both the center frequency and bandwidth decrease to about 15 Hz and 5–10 Hz, respectively, accompanied by an increased occurrence of sleep spindles. As sleep deepens into the N2 stage, the center frequency further declines to around 5 Hz, the bandwidth narrows to 5 Hz, and the amplitude at the central frequency becomes more pronounced [43]. In the N3 stage, the center frequency approaches 0 Hz, the bandwidth remains consistent with N2 at around 5 Hz, but the amplitude at the central frequency increases substantially, typically ranging from 150 to 400 V^2^/Hz [43]. Band energy analysis showed that alpha and beta energy are highest during wakefulness and lowest during the N3 stage, while theta energy peaks in the REM stage, and delta energy is most dominant in the N3 stage [13]. This pattern shifts under sleep deprivation, where relative theta and delta increase, and alpha/beta decrease [44].

Additionally, the spectral slope, which is steeper during the REM stage, reflects enhanced cortical inhibition and tends to flatten with age, serving as a potential biomarker for sleep health [45]. Also, the Spectral Edge Frequency difference (SEFd)—defined as the difference between SEF95 and SEF50 in 2 s sub-epochs—showed consistent peaks in the REM stage and lower values in N2 and N3 stages, with slight fluctuations in N1. Finally, Absolute Power (AP) in the 8–16 Hz range was lowest in the REM stage and higher in the Wake and N1 stages, supporting REM stage classification [46].

To complement spectral and statistical descriptors, non-linear features were extracted to evaluate signal irregularity and complexity. Spectral Entropy, Singular Value Decomposition (SVD) entropy, Lempel–Ziv Complexity (LZC), and Rényi Entropy were calculated, which are particularly relevant for assessing brain dynamics in stress and sleep pathology [14,15]. LZC tends to be elevated under stress, particularly in frontal sites (Fp1/Fp2), reflecting increased cortical activity. In contrast, Rényi entropy decreases under similar conditions, indicating reduced global brain flexibility and increased rigidity [40].

Therefore, an initial pool of 100 features was defined through a systematic review of the literature on EEG-based sleep staging, with a focus on features that have consistently demonstrated high correlation with sleep stage transitions. These included canonical time-domain measures (e.g., amplitude statistics, Hjorth parameters), frequency-domain metrics (e.g., bandpower in δ, θ, α, σ, β ranges), and selected non-linear descriptors (e.g., entropy measures). Starting with a validated and physiologically meaningful feature set ensured that the dimensionality reduction and subsequent modeling steps were grounded in features most relevant to sleep physiology, rather than an arbitrarily large or unstructured feature space.

Tests on sleep-acquired signals and extraction of the described features for sleep staging were conducted using the BOAS dataset; it was gathered within a project aimed at bridging the gap between gold-standard clinical sleep monitoring using the Micromed Brain Quick Plus Evolution PSG system (manufactured by Micromed S.p.A., Mogliano Veneto, Italy) and emerging wearable EEG technologies, specifically the Bitbrain EEG headband (https://openneuro.org/datasets/ds005555/versions/1.0.0) (accessed on 3 February 2025). The dataset includes 128 nights of simultaneous recordings from both systems in healthy participants. The PSG system provides a comprehensive, clinically validated set of sleep parameters, while the Bitbrain wearable EEG headband offers a user-friendly, self-administered alternative limited to forehead EEG electrodes. The simultaneous data acquisition allows for direct comparison and validation of the wearable EEG device against the established PSG standard, creating a valuable resource for evaluating the performance and potential of wearable EEG technology in sleep studies. A rigorous labeling process was employed to ensure robust and reliable sleep staging. Three expert sleep scorers independently annotated the PSG recordings according to the AASM criteria [47]. A fourth expert derived consensus labels from these annotations to address the inherent variability in human sleep staging, which has an estimated inter-scorer agreement of approximately 85% [48,49]. These consensus labels were then applied to the corresponding wearable EEG recordings, leveraging the simultaneous data acquisition. A deep learning model [50] was utilized to analyze the dataset; the tests of the model for single-channel sleep staging showed that the Wake, N2, and N3 classes achieved values of accuracy, precision, recall, and F1-score of approximately 80%, while the REM classification fell to around 70% in all metrics. Nevertheless, the N1 phase was most prone to misclassification, with accuracy decreasing to 37% [50]. Using a cross-validation procedure, the model was trained and validated separately on the PSG and wearable EEG datasets. It achieved an 87.08% match between human-consensus labels and model-predicted labels for the PSG data and an 86.64% match for the wearable EEG data [51]. These results highlight the potential of wearable EEG technology to approximate the accuracy of traditional PSG systems, offering a promising alternative for sleep monitoring in clinical and non-clinical settings. The study highlights the importance of rigorous validation and consensus labeling in ensuring the reliability of sleep staging and the effectiveness of machine learning models in advancing sleep research. In this study, rather than using model-derived annotations, we employed annotations established by consensus among the physicians for feature selection and model development.

To evaluate the effectiveness of selected EEG features in sleep stage classification, recordings corresponding to the F4–F3 derivation were randomly chosen from the labeled sleep dataset described. The data were preprocessed with a 0.2–44 Hz bandpass filter to suppress irrelevant frequency components and a 50 Hz notch filter to remove power line interference. The cut-off frequencies of the bandpass filter were selected to retain the conventional EEG frequency range that is physiologically relevant for sleep staging, encompassing delta, theta, alpha, sigma/spindle, and beta activity, while attenuating slow drifts and high-frequency noise. The 0.2 Hz lower cut-off was chosen to minimize baseline wander due to respiration, motion, or electrode impedance fluctuations, without compromising the slow oscillations (<1 Hz) that are characteristic of NREM sleep. The 44 Hz upper cut-off was set to eliminate muscle artifacts and environmental noise while preserving the beta range (up to ~30 Hz) that may contribute to sleep stage discrimination. Higher frequencies (>44 Hz) were excluded as an acceptable trade-off to improve the signal quality and generalizability of the method in wearable applications. To minimize the risk of phase distortion and delay, a zero-phase forward–backward IIR implementation was employed, which effectively cancels phase shifts by applying the filter in both forward and reverse directions. This approach preserves the temporal alignment of EEG waveforms, which is critical for sleep staging and feature extraction, particularly when analyzing spindle activity or slow oscillations. However, for processing signals in non-clinical settings, algorithms for removing eye movements, muscular contractions, and ECG artifacts, such as linear regression or ICA-based filtering, will be investigated.

The filtered signals were segmented into non-overlapping 5 s windows, with each segment labeled according to the corresponding 30 s sleep stage in the BOAS dataset. Feature extraction was carried out using MATLAB software (version 24.1), followed by feature selection using the mRMR algorithm.

**Figure 3:**
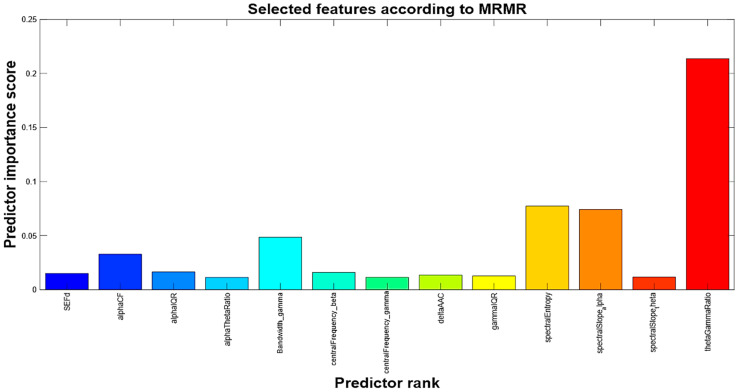
Rank of features obtained by the mRMR algorithm with a score higher than 0.01.

The mRMR algorithm is a widely used feature selection technique that identifies a compact and informative subset of features by simultaneously maximizing relevance to the target variable and minimizing redundancy among the features themselves. Relevance is assessed through Mutual Information (MI), which quantifies the statistical dependency between an input feature and the class label, ensuring that each selected feature contributes unique information for classification tasks. The aim is to find the optimal subset of features (S), maximizing the relevance of S (V_S_) concerning the target variable (y) and minimizing the redundancy of S (W_S_):

(2) $$V_{S}=\frac{1}{\left|S\right|}\sum_{x_{i}\in S}I(X_{i},\;y)MIQ_{x}=\frac{V_{x}}{W_{x}}$$

(3) $$MIQ_{x}W_{S}=\frac{1}{{\left|S\right|}^{2}}\sum_{x_{i},z\in S}I(X_{i},z)$$

where $\left|S\right|,$ is the number of features in S and I( , ), the mutual information. The selection process follows an iterative approach, initially selecting the feature with the highest relevance and subsequently incorporating features that optimize the Mutual Information Quotient, defined as the ratio of relevance to redundancy:

(4) $$MIQ_{x}=\frac{V_{x}}{W_{x}}$$

where $V_{x}$ and $W_{x}$, the relevance and redundancy of x features. By prioritizing features that provide complementary and non-redundant information, mRMR enhances model interpretability and efficiency, making it particularly advantageous for high-dimensional datasets such as EEG signal analysis [52,53]. Owing to these characteristics, the mRMR algorithm was adopted in the present study to evaluate and rank features based on their discriminative relevance and mutual redundancy, thereby facilitating the selection of the most informative and non-redundant subset for sleep stage classification. This ranking was derived from EEG recordings of 7 randomly selected subjects (indicated by 1, 5, 10, 25, 55, 75, and 100), covering ≈56 h of sleep (≈8 h of recording for each subject) and producing 42,858 five-second segments. By applying a relevance score threshold of 0.01, the initial set of 100 features was reduced to 13 top-performing features, as shown in Figure 3. To further streamline the dataset and enhance computational efficiency, PCA was applied.

**Figure 4:**
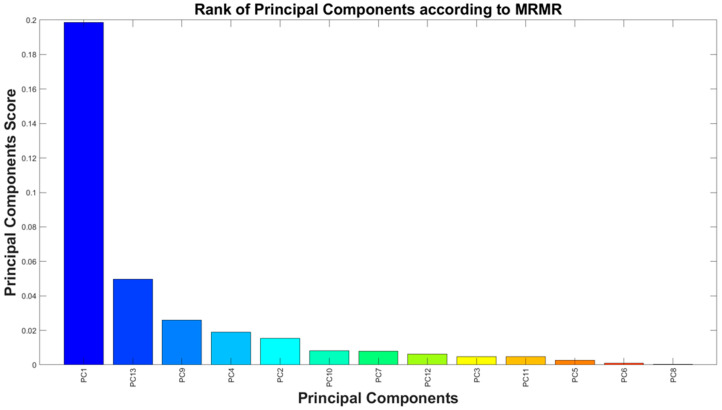
Rank of Principal Components by the mRMR algorithm.

In datasets with many variables, it is common for some of them to measure the same underlying principle governing the system’s behavior, leading to redundancy. PCA addresses this by transforming the original variables into a set of mutually orthogonal principal components, each representing a linear combination of the initial features. The first component captures the maximum variance, with each subsequent component accounting for the next highest variance while remaining orthogonal to the others. Although all components together retain the original dimensionality, PCA enables dimensionality reduction by selecting only those that explain most of the variance—typically assessed via the explained variance ratio. By reducing redundancy and preserving as much information as possible, PCA simplifies data analysis, making it a powerful tool for feature selection, noise reduction, and visualization in high-dimensional datasets. Given the potential redundancy even within a reduced feature space, PCA was applied to decorrelate the selected features and optimize their representation. With cumulative variance thresholds of 98% and 94%, 13 and 11 PCs were retained, respectively, effectively capturing the most critical information from the original features. Figure 4 illustrates the ranking of the 13 PCs according to their importance scores obtained with the mRMR. Each bar corresponds to a specific principal component extracted from the PCA transformation of the feature set, and the height of the bar represents the relevance score assigned by mRMR. The components are ordered in descending order of importance, meaning the leftmost bars indicate the PCs that carry the most discriminative power for distinguishing between the sleep stages in the dataset. Table 3 summarizes the explained and cumulative variance of each principal component. As can be noticed, applying a 94% variance threshold, an optimal subset of 11 PCs was identified (highlighted in green). This optimized set of components was then used to train machine learning models for sleep staging based on a single EEG channel. The 94% threshold was chosen as a balance between maximizing information retention and minimizing feature dimensionality.

Table 4 summarizes the previously selected features, categorized by analysis objective: sleep staging, assessment of sleep deprivation conditions, and evaluation of psychological stress. Sleep deprivation occurs when insufficient or inadequate nighttime rest compromises the normal physiological recovery processes. This state can result from reduced total sleep duration, sleep fragmentation, or alterations in normal sleep architecture, negatively affecting cognitive and bodily functions. For example, significant variations in EEG signal power are observed in individuals with severe sleep deprivation, depending on the duration and quality of their recovery sleep cycle. Specifically, the power of slow waves is higher in the second cycle than in the first, while the power of alpha waves is higher in the first cycle than in the second [54]. Psychological stress is a state of stress detectable in EEG signals caused by sleep deprivation, which can be identified through sleep staging analysis or by examining specific EEG features. Among these, nonlinear-domain EEG features are particularly useful since they provide additional information that enhances the ability to distinguish sleep stages and, more generally, different psycho-physiological states [14].

In our work, the features were extracted in an offline manner; in principle, real-time implementation could introduce differences relative to offline analysis due to constraints such as limited buffer sizes, streaming delays, or computational resources. However, the preprocessing and feature extraction steps employed in this study were designed with real-time feasibility in mind. The bandpass and notch filters can be implemented as causal, low-order digital filters with negligible delay when applied in a streaming context. The selected features (time, frequency, and non-linear metrics) rely on computationally efficient operations (e.g., windowed FFTs, entropy measures) that are compatible with real-time execution. Moreover, since our dimensionality reduction strategy (mRMR + PCA) operates on a fixed feature space, it can be easily applied to an incoming data stream, extracting the features selected by mRMR and calculating the PCs on the considered 5s epoch. At the same time, offline analysis allows for zero-phase filtering (e.g., forward–backward filtering) and batch-level artifact rejection; these differences are not expected to significantly degrade performance in the context of sleep staging, where the temporal resolution is on the order of 5 s epochs.

## 4. Results

### 4.1. Experimental Tests on the Acquisition of the EEG from the Forehead, Ear, and In-Ear

This section presents the analysis of the experimental results related to the acquisition and processing of EEG signals recorded using the previously described setup, considering the four selected derivations and the experimental conditions defined in the previous Section 3: “open eyes”, “closed eyes”, “evoked1”, and “evoked2”.

**Figure 5:**
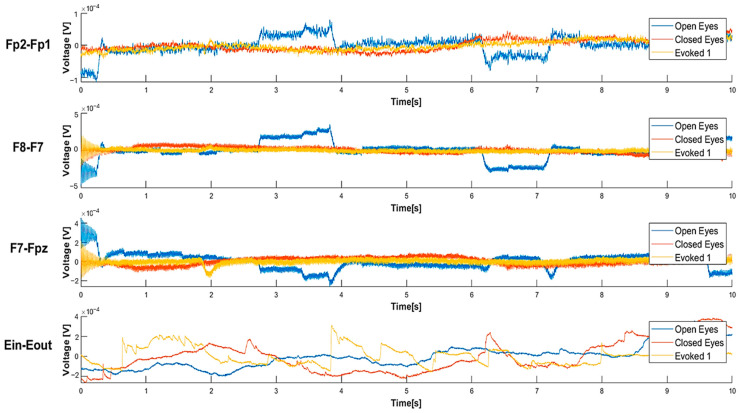
Comparison of waveforms acquired in different conditions (i.e., open eyes, closed eyes, evoked1) for different EEG derivations (Fp2-Fp1, F8-F7, F7-Fpz, and Ein-Eout).

Figure 5 shows that, in the “open eyes” condition, all the considered derivations exhibit a high amplitude of low-frequency waves. In the “closed eyes” condition, the derivations acquired from the forehead significantly reduce the amplitude of slow waves. However, the Ein-Eout derivation shows the presence of slow waves with considerable amplitude even in the “closed eyes” and evoked conditions. Analyzing the absolute power values, it is observed that the Fp2-Fp1 derivation presents a high-power value in the “open eyes” condition, which significantly decreases in the “closed eyes” and evoked conditions (Table 5).

Similarly, in the F8-F7 derivation, a reduction in absolute power is recorded when transitioning from the “open eyes” to the “closed eyes” condition; in contrast, the power value is higher in the VEP condition than in the “closed eyes” condition. For the F7-Fpz derivation, power is higher in the “open eyes” condition, while in the “closed eyes” and evoked conditions, it remains in the same order of magnitude. Finally, for the Ein-Eout derivation, the maximum power is recorded in the “open eyes” condition and decreases in the other two conditions. Depending on the experimental condition, these variations can be attributed to a change in the power distribution among the different wave components. Specifically, the transition from the “open eyes” to the “closed eyes” condition reduces the power of slow waves, which are characterized by higher amplitude, in favor of higher-frequency waves, which exhibit lower amplitude. This phenomenon results in a decrease in the absolute power of the EEG signal.

**Figure 6:**
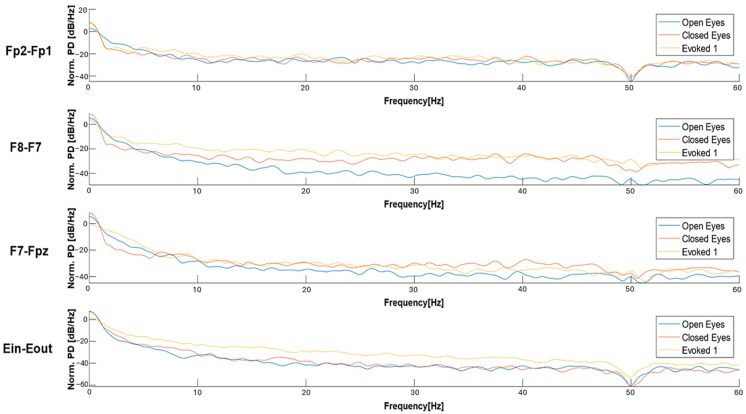
Comparison of the normalized PSDs of EEG signals acquired in different conditions (i.e., open eyes, closed eyes, evoked1) for the four considered derivations: Fp2-Fp1, F8-F7, F7-Fpz, and Ein-Eout.

Figure 6 compares the power spectral density (PSD) of EEG signals for the four derivations in the three conditions. For the Fp2-Fp1 derivation, an increase in relative power is observed in the alpha waves and, more generally, in the higher frequencies in the “closed eyes” and evoked conditions compared to the “open eyes” condition. For the F8-F7 derivation, transitioning from the “open eyes” to the “closed eyes” and evoked conditions significantly increases power at higher frequencies. In particular, in the evoked condition, a greater distribution of power in the alpha and beta bands is observed, which can be attributed to a sensory activation state induced by the visual stimulus. Similar trends are also found in the F7-Fpz derivation. Additionally, for the Ein-Eout derivation, the evoked condition shows a significantly higher power spectral density than the “open eyes” and “closed eyes” conditions, ranging from theta waves to higher frequencies.

**Figure 7:**
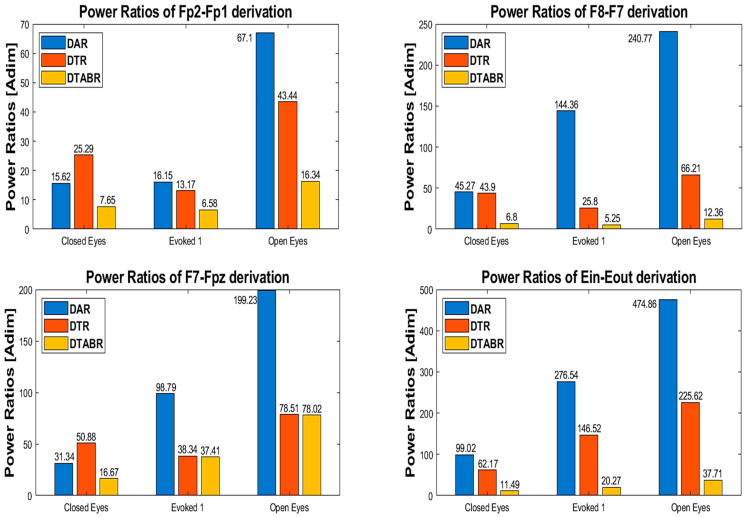
Power ratios of the four derivations (Fp2-Fp1, F8-F7, F7-Fpz, Ein-Eout) in three conditions (open eyes, closed eyes, evoked1).

Figure 7 presents a bar graph displaying the values of the delta–alpha ratio (DAR), delta–theta ratio (DTR), and delta–theta–alpha–beta ratio (DTABR) for the four derivations. These power ratios, calculated from the power of the EEG signal in different frequency bands, are defined as follows:

- The DAR is the ratio of delta wave power to alpha wave power.
- The DTR is the ratio of delta wave power to theta wave power.
- The DTABR is defined as the ratio of the sum of delta and theta wave power (slow waves) to the sum of alpha and beta wave power (fast waves).

While the experimental tests conducted with the custom acquisition setup demonstrated the feasibility and quality of EEG recordings from both forehead and in-ear positions, the limited sample size and controlled laboratory conditions were not sufficient for comprehensive feature evaluation and model training. For this reason, the subsequent analysis of feature relevance and classification performance was conducted using the BOAS dataset, which provides a large volume of well-annotated polysomnography-acquired EEG signals from multiple subjects. Leveraging this clinically validated dataset ensured statistical robustness, reproducibility, and a more reliable benchmark for evaluating algorithms, while our custom setup served primarily to validate the practical applicability of wearable EEG acquisition in real-world scenarios.

### 4.2. Training and Testing of Sleep Staging Algorithms

This section presents the training and evaluation outcomes of the proposed two-step sleep staging algorithm, developed using both the complete and reduced feature sets. The approach is based on two sequential LSTM-based models consisting of an LSTM and dense layers, each designed for a 3-class classification task and together providing a comprehensive 5-class sleep staging output. The first model classifies EEG epochs into Wake, REM, and NREM stages, leveraging the LSTM’s capability to capture temporal dependencies inherent in physiological signals. The second model is dedicated to refining the NREM classification by distinguishing between its substages—N1, N2, N3—focusing on the subtle signal variations that differentiate transitional, light, and deep sleep within the NREM spectrum. Following feature scaling, the dataset was partitioned into stratified training (80%) and test (20%) subsets. Model development and validation were conducted within the training set using a stratified five-fold cross-validation scheme, thereby ensuring rigorous performance estimation and mitigating bias arising from sample variability. The employed strategy prevents data leakage, ensuring class balance in both splits, and provides both reliable training and validation, as well as an independent test performance measure. Class weights were computed to address class imbalance, with the minority class corresponding to REM sleep further upweighted to improve sensitivity. The target labels were one-hot encoded for compatibility with categorical cross-entropy loss. The model architectures were based on an attention-augmented LSTM network, which received as input the time-series feature matrix and predicted one of three classes. In particular, both classification models were implemented as a recurrent neural network with an attention mechanism. The input to the network was a multivariate time series of shape (timesteps × features). The first recurrent block consisted of a 128-unit LSTM layer with L2 regularization, followed by batch normalization. An attention mechanism was then applied: a dense layer with tanh activation generated attention scores, which were normalized via softmax and used to weight the hidden representations of the LSTM outputs. The weighted sequence representation was processed by a second LSTM layer with 64 units, also with L2 regularization, followed by batch normalization and a dropout layer (dropout rate = 0.3) to mitigate overfitting. The resulting feature vector was passed through a 64-unit fully connected layer with ReLU activation and an additional dropout layer (dropout rate = 0.2). Finally, the output layer employed a softmax activation to produce class probabilities over the specified number of classes. The loss function employed was a weighted categorical cross-entropy to reflect the class weighting scheme. Moreover, the training of each classifier lasts 50 epochs using the AdamW optimizer, with a learning rate of 0.0005 and a weight decay of 0.0001.

**Figure 8:**
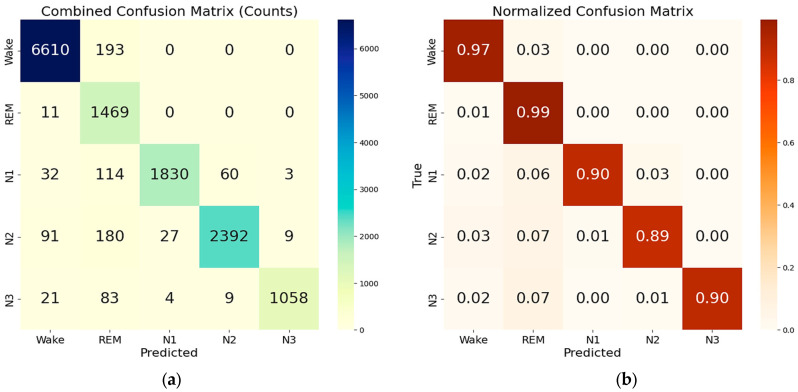

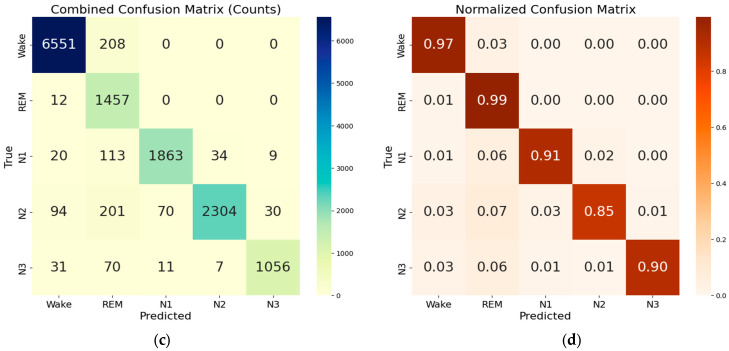
Confusion matrices and normalized confusion matrices for the 5-class sleep classifier using the two three-class LSTM models developed employing reduced feature sets: 98% (13 features) (**a**,**b**) and 94% (11 features) (**c**,**d**) threshold on the cumulative explained variance of PCs.

The combined two-step LSTM-based model demonstrated strong performance across both reduced and complete feature sets. When using the reduced feature set, the model achieved an overall accuracy of 94.7% downstream of the model test, with a macro-average F1-score of 0.932 and a weighted average F1-score of 0.949 (Table 6). Class-wise, the model maintained balanced and reliable predictions, particularly excelling in the Wake (F1-score: 0.977) and deep sleep (N3: 0.953) stages. REM, often challenging due to its physiological overlap with other stages, was still classified with an F1-score of 0.852 and a recall of 0.991, indicating a high sensitivity to REM episodes. The corresponding confusion and normalized confusion matrices for this configuration evaluated on the test set are presented in Figure 8a,b. The model also demonstrated excellent memory efficiency, a crucial requirement for deployment in low-power or embedded environments. The memory profile throughout the evaluation pipeline remained consistently low. The process began with an initial memory allocation of 186.32 MB, which increased to 376.21 MB after the data was loaded. Following the WRN (Wake–REM–NREM) stage prediction, memory usage slightly decreased to 372.67 MB. After adjusting for sample mismatches between WRN and NREM stages, the model completed the NREM classification stage and result assembly with a final memory footprint of 373.23 MB.

As previously discussed, to further reduce the dimensionality of the feature set, the threshold on the cumulative explained variance of the principal components (PCs) derived from the PCA was reduced to 94%, resulting in a feature set constituted by 11 features (PCs) (Table 3). The resulting feature set was used to train and test the same two-step ensemble model previously described. The confusion matrices of the combined model for 5-class classifications, evaluated on the test set, are reported in Figure 8c,d, and the resulting performances are summarized in Table 7.

The reduction in the feature set impacts the model’s performance, as evidenced by a decrease in overall accuracy to 93.6% (−1.2%) compared to the previous feature set. In detail, the classes primarily affected by the reduction in feature set dimensionality are REM, N1, and N3. The drop in REM precision (−4.8%) indicates a degradation in the model’s ability to distinguish between non-REM and REM samples. Similarly, this degradation is evident in the N3 stage, where the precision decreases from 99.2% to 96.4% (−2.8%).

Also, reducing the feature set dimensionality results in a reduction in memory requirements, which is 338.39 MB after building results (−9.3% compared to the previous feature set). Specifically, the model requires 123.11 MB of initial memory, which increases to 301.11 MB after loading the data and reaches 338.39 MB after building the results.

**Figure 9:**
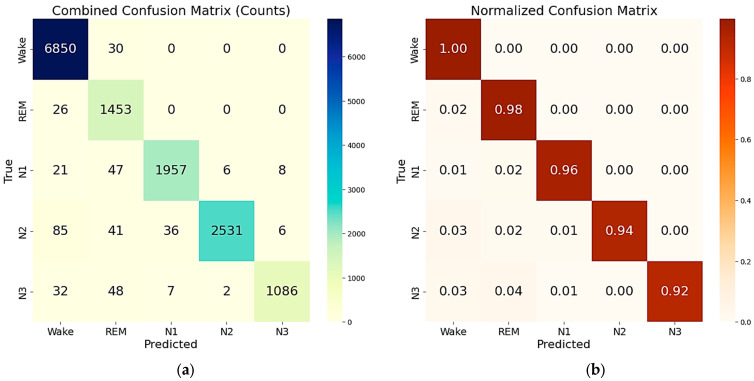
Confusion (**a**) and normalized confusion (**b**) matrices for the 5-class sleep classifier using the complete feature set.

In contrast, when trained and evaluated on the complete feature set (comprising 100 features), the two-step model achieved an overall accuracy of 97.9%, significantly improving all key metrics. The related confusion and normalized confusion matrices are shown in Figure 9, and detailed performance metrics are presented in Table 8. The macro-average and weighted average F1-scores reached 0.969 and 0.979, respectively. All classes exhibited high classification performance, with F1-scores ranging from 0.934 (REM) to 0.993 (Wake). The precision and recall for each stage remained high, demonstrating robust and reliable identification across all sleep stages. However, this improvement came with a higher memory footprint of 573.05 MB after the result assembly and confusion matrix generation were completed, which may be less suitable for constrained environments.

Nevertheless, these gains came at the cost of a substantially higher memory footprint, making this configuration less ideal for constrained environments such as embedded or mobile systems. The initial memory allocation was 234.95 MB before loading the data. Once the data was loaded, memory usage increased to 563.71 MB. After running predictions for the first stage (Wake–REM–NREM classification), memory usage rose to 571.02 MB. Following adjustments for sample alignment between WRN and NREM stages, and executing the second-stage predictions, memory usage peaked at 572.69 MB. Finally, during result assembly and confusion matrix generation, memory usage reached 573.05 MB.

Overall, the results demonstrate that the two-step model achieves excellent performance with both reduced and complete feature sets, each offering advantages suited to specific application needs. The reduced feature set provides a memory-efficient solution with 94.7% accuracy (only 3.2% lower than that achieved with the full feature set), making it ideal for real-time, resource-constrained applications. With a memory usage of only 373.2 MB, this configuration is well-suited for real-time sleep staging, embedded systems, mobile health devices, and edge computing platforms, where computational resources are limited. Meanwhile, the complete feature set maximizes the classification accuracy (97.9%), but at the cost of over 50% higher memory usage (573.1 MB, related to the final step of result assembly and confusion matrix generation). While the complete feature set yields the highest classification performance, its memory demand suggests that it is best suited for performance-critical offline analysis or resource-rich environments, rather than low-power, real-time applications.

## 5. Discussions

### 5.1. Feature Insights and Trends from Acquired EEG Signals

**Figure 10:**
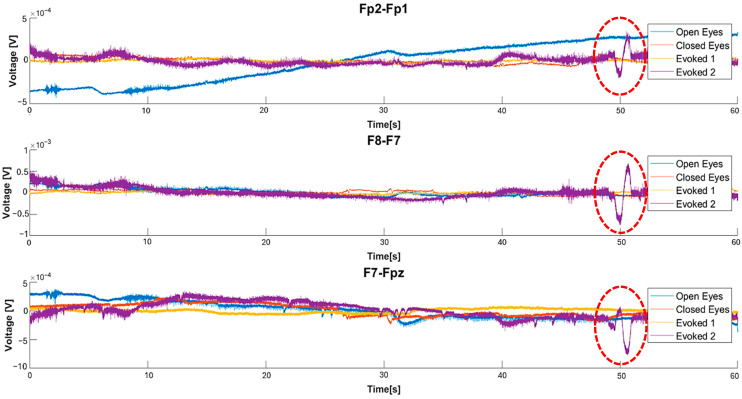
Waveforms’ comparison in the different conditions (open and eyes, evoked1, evoked2) for the Fp2-Fp1, F8-F7, and F7-Fpz derivations. An artifact is highlighted in red due to eye muscle’s contraction or spasm.

The features identified were extracted from 60-s signals acquired using the experimental setup described in Section 3, considering only frontal derivations, and then filtered by a 50 Hz notch filter to remove power-line interference. Figure 10 presents a comparison of waveforms in different conditions for each derivation, highlighting increased EEG activity in the evoked conditions featuring audio-visual stimuli. A significant increase in amplitude of high-frequency components is observed compared to other conditions across all three derivations; furthermore, the “evoked2” condition shows a stronger response than “evoked1,” as expected, due to the higher intensity of the applied stimulation.

**Figure 11:**
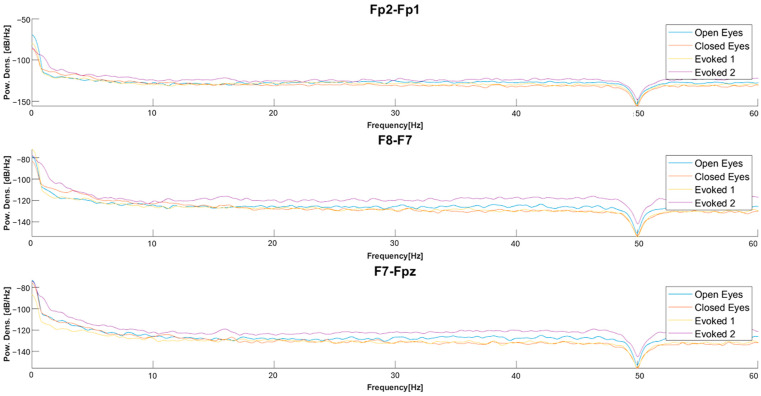
Comparison of the PSDs of the EEG signal acquired in different conditions (i.e., “open eyes”, “closed eyes”, “evoked1”, and “evoked2”) related to Fp2-Fp1, F8-F7, and F7-Fpz derivations.

The comparison of the PSDs in Figure 11 suggests that, in the “open eyes” condition, the power is lower for high-frequency contributions and higher for low-frequency ones. In the “evoked1” condition, a power reduction in the low-frequency components and an increase in high-frequency components are observed. Specifically, the power spectral density in “evoked1” is lower compared to the “open eyes” and “closed eyes” conditions in the low-frequency range. This trend is also confirmed in the “evoked2” condition, where significant components remain in the 0.5–4 Hz range. Previous studies have reported that exposure to emotional visual stimuli significantly increases delta wave power, with the amplitude of these oscillations modulated by the level of emotional involvement [61,62].

After, the acquired signals were segmented into 5 s windows with a 30% overlap, and features were extracted from each window to identify a correlation between their values and the tested conditions. Signal segmentation increased the number of extracted windows, thereby extending the dataset and ensuring that each window contained an equal number of samples. Specifically, the implemented segmentation scheme ensures that the last window is completed with samples from before its start, thereby matching the sample count of the other windows. The results obtained from comparing the four experimental conditions enable a comprehensive evaluation of the extracted features, allowing for their validation even in the presence of stress-like conditions. Table 9 shows the mean value of the features for the “open eyes”, “closed eyes”, “evoked1”, and “evoked2” conditions.

As regards the signals acquired by the experimental setup, the power ratio analysis shown in Figure 7 reveals a progressive decrease in the DAR from “open eyes” to “closed eyes” and evoked conditions. For frontal derivations, a DTR ratio reduction is recorded from “closed eyes” to evoked, which is not observed for the in-ear derivation. Finally, DTABR decreases when transitioning from “open eyes” to “closed eyes” due to the decrease in delta wave power and increase in fast wave power; instead, when moving from “closed eyes” to evoked, DTABR increases likely due to a greater theta wave power induced by visual stimuli.

Observing the power ratio values reported in Figure 7, it is clear that, for the same condition, the DAR and DTR power ratios assume higher values for the (Eout-Ein) derivation compared to signals acquired from frontal derivations. This result can be attributed to the greater power of the low-frequency components falling within the delta band compared to those in other frequency bands. However, the previously discussed trends regarding the variation in power ratios across different conditions remain unchanged for the auricular derivation, demonstrating the capability of the developed in-ear EEG acquisition system to capture similar information conveyed by signals from the forehead.

The validation of EEG-based features extracted in the time domain is based on several observations that confirm their reliability. Regarding Hjorth parameters, activity corresponds to variance and is therefore greater in the “evoked2” condition than in the “eyes open” one. Moreover, complexity is defined as the ratio between the mobility of the signal’s derivative concerning time and the mobility of the signal itself; therefore, since the signal recorded in the “open eyes” condition has lower mobility compared to “evoked2”, complexity is higher in the “open eyes” condition compared to the “evoked2” condition.

The following checks were performed to validate the reliability of spectral features; as expected, the low-frequency waves exhibit greater power than the high-frequency ones, since their amplitude is higher. In some windows, the relative power of beta and gamma waves is higher than that of theta and alpha, likely due to some spikes or artifacts; however, generally, the power of low-frequency waves is greater than that of high-frequency waves. Therefore, the power ratios between slow wave power and high-frequency components (e.g., DAR, DTABR) result in values greater than 1, aligning with the greater power of low-frequency waves. In addition, the Delta Slow Wave Index (DSI) is significantly larger than ASI and Theta Slow Wave Index (TSI), as higher power is distributed in the delta band. Finally, the spectral slope is generally negative, given the shape of the EEG signal’s PSD, with a rapid decrease as the frequency increases. Furthermore, the absolute value of the slope tends to decrease as the frequency moves toward higher bands. In some cases, it takes positive values, reflecting the typical behavior of the EEG signal’s PSD, which shows a rapid decrease at low frequencies and a flattening at higher ones.

The reliability of the non-linear features extracted from the EEG signal can be validated by analyzing the variation in parameters between two conditions: the “open eyes” condition, considered as a reference, and the “evoked2” condition, associated with a state of stress-like condition. Regarding the Rényi entropy, the literature suggests a lower value in the “evoked2” condition compared to the “open eyes” condition [43]. This trend reflects a decrease in the global complexity of brain dynamics, consistent with a more rigid and less flexible brain system under stress. This phenomenon is confirmed by the extracted data: in the “evoked2” condition, the mean value of the Rényi entropy is 2.4628, whereas in the “open eyes” condition, it is 2.7372. Furthermore, it is well established that stress affects neuronal activity, making the EEG signal more complex and less predictable. Consequently, SVD Entropy tends to increase in stressful situations. The collected data confirm this behavior; in the “evoked2” condition, the mean SVD Entropy value is 0.6629, while in the “open eyes” condition, it equals 0.2509. Spectral entropy also follows a similar trend; in the “open eyes” condition, it has a 0.1393 mean value, while in the “evoked2” condition, there is an increase, with a mean value of 0.4123.

Although it is not considered a non-linear parameter, the RSP values in the alpha band, according to the ref. [43], can be compared between the two considered conditions. The study indicates a significant increase in the values of this feature in stressed subjects, with greater involvement of the right hemisphere (Fp2) associated with greater activation under stress. In this case, as well, the results confirm expectations; in the “open eyes” condition, the mean alpha band RSP is 8.4320 × 10^−3^, while in the “evoked2” condition, an increase is observed, with a mean value equal to 3.3350 × 10^−2^. These observations demonstrate a general consistency between the analyzed parameters and the expected results, indicating the validity of the calculated features under the experimental conditions.

Overall, the experimental EEG acquisitions demonstrate that the reduction in slow-wave amplitude in forehead derivations during the transition from open to closed eyes reflects the expected increase in alpha rhythm, a well-documented physiological response to reduced visual input and cortical idling. Also, the persistence of slow-wave activity in the in-ear derivations suggests that these channels may be more sensitive to deeper subcortical or mixed sources, which could be clinically relevant in scenarios where forehead recordings are less stable or more prone to artifacts. The higher alpha and beta power during evoked conditions are consistent with stimulus-driven sensory activation, supporting the ability of the proposed setup to capture physiologically meaningful reactivity.

As part of the SOMNIIA MONITOR research project, we have begun collecting a dataset considering the derivations above during sleep in healthy patients. The creation of a proprietary dataset will allow us to validate the developed model in conjunction with the proposed setup, enabling us to test different electrode configurations and optimize signal acquisition for accurate sleep stage detection.

### 5.2. Analysis of Sleep Stage Variability Using the Coefficient of Variation Metrics

Afterwards, the variability over time of the selected feature set was analyzed to quantify physiological variability across the complete spectrum of sleep–wake states through advanced statistical analysis of feature data extracted from polysomnographic recordings. The analysis encompassed a comprehensive dataset comprising 42,858 distinct 5 s temporal windows, each characterized by 100 multidimensional features representing various physiological domains, including electrophysiological signals, autonomic nervous system activity, and movement-derived parameters.

This study conducted a comprehensive analysis of physiological variability across five distinct sleep stages using the absolute Coefficient of Variation (CV) measurements derived from 5 s window feature data. Table 10 presents the comprehensive variability analysis across sleep stages using absolute CV metrics, revealing distinct patterns of physiological fluctuation throughout the sleep–wake cycle.

The analysis revealed a clear gradient of variability that aligns with established principles of sleep physiology. Wakefulness demonstrated the highest median CV (125.05%), reflecting the expected physiological instability during conscious states characterized by frequent movements, cognitive transitions, and environmental interactions. Following wakefulness, N2 sleep exhibited substantial variability (101.07%), consistent with its dynamic nature, which features sleep spindles, K-complexes, and periodic arousal fluctuations. N1 sleep, representing the transitional light sleep phase, exhibited moderate variability (93.11%), capturing its unstable characteristics as subjects move between wakefulness and deeper sleep stages. REM sleep exhibited lower variability (87.20%), indicating paradoxical yet relatively stable cortical activation patterns, despite the presence of rapid eye movements. Most notably, N3 deep sleep demonstrated the lowest variability (71.56%), underscoring the profound stability and synchronization of slow-wave activity characteristic of this restorative sleep stage. These findings robustly demonstrate that sleep architecture follows a hierarchical pattern of variability, where conscious and light sleep stages exhibit greater physiological fluctuation, while deeper sleep stages exhibit increasing stability.

### 5.3. Development of a Deep Learning Algorithm for Sleep Staging

The increasing demand for portable and non-invasive sleep monitoring technologies has driven the advancement of EEG-based systems that aim to reduce hardware complexity while maintaining diagnostic accuracy. With the growing emphasis on efficient sleep analysis, deep learning models tailored for sequential data—particularly LSTM networks—have shown significant capability in modeling the temporal characteristics of EEG signals. This study introduces a two-stage deep learning framework that leverages these capabilities to perform five-class sleep staging using a reduced set of frontal EEG channels, offering a promising solution for integration into wearable and low-power embedded systems.

The overall performance of the proposed sleep staging framework, which cascades two 3-class classifiers, confirms that reliable multi-class classification can be achieved using only frontal EEG signals. Achieving an overall accuracy of 94.7% and a macro-average F1-score of 0.932—using the reduced feature set obtained via mRMR and PCA—highlights the effectiveness of the two-step approach, even while using a reduced feature set. Specifically, the model exhibited excellent detection of the Wake stage, with an F1-score of 0.977, driven by high precision (0.979) and recall (0.974). The REM stage, often challenging due to its physiological similarities with other stages, exhibited strong and consistent classification outcomes, reaching an F1-score of 0.852 and a recall of 0.991, although precision was relatively lower (0.747), indicating some false positives. Among the NREM sub-stages, N1 achieved an F1-score of 0.946 with remarkably high precision (0.993) but slightly lower recall (0.903), likely due to its transitional overlap with N2. The N2 stage, comprising the largest dataset portion, achieved a score of 0.934 (F1), supported by a precision and a recall of 0.967 and 0.902, respectively. The N3 stage maintained strong performance with an F1-score of 0.953, reflecting high precision (0.992) and recall (0.917). These results underscore that even a single-channel frontal EEG, when paired with optimized feature selection and temporal modeling via LSTM networks, can achieve detailed and reliable sleep staging.

Afterward, the impact of more stringent dimensionality reduction was evaluated by applying the PCA with a 94% cumulative variance threshold, which reduced the feature set from 13 to 11 PCs. The further dimensionality reduction in the feature set results in a slight decrease in overall accuracy (93.6%) compared to that obtained with 13 PCs (94.7%). This slight performance reduction (−1.2%) can be considered a worthwhile price to pay to achieve a reduction in memory requirements. Using the reduced feature set (13 features), the complete classification pipeline maintained a low memory footprint, peaking at 376.21 MB; the reduction in feature set dimension (11 features) further improved memory efficiency, lowering the model’s peak memory usage to 338.39 MB (−9.3%), making the proposed model ideal for real-time applications on embedded systems and wearable platforms. Similarly, the F1-score follows the same trend, decreasing from 0.949 (weighted average) to 0.938 (−1.2%) as the feature set dimensionality is reduced from 13 to 11. From the point of view of the performance of the single classes, the reduction in feature set dimensionality has the most pronounced effect on the REM, N1, and N3 sleep stages. As for REM, the precision decreases by 4.8%, indicating that the model becomes less effective at distinguishing between REM epochs and non-REM ones, which is particularly critical given the physiological importance of REM sleep. The N3 stage also exhibits a notable decline, with precision dropping from 99.2% to 96.4% (−2.8%), indicating a reduced ability to identify deep sleep reliably. Although the impact on N1 is less severe, the observed degradation highlights that stages characterized by either transitional features (N1) or high specificity (REM, N3) are more sensitive to dimensionality reduction. Finally, the obtained results suggest that a trade-off exists between model accuracy and memory usage, which can be exploited according to the constraints imposed by the employed hardware.

In contrast, using the complete feature set (100 features) resulted in a noticeable increase in classification performance, achieving an overall accuracy of 97.9%, with macro and weighted F1-scores of 0.969 and 0.979, respectively. All sleep stages exhibited excellent classification performance under this configuration, with F1-scores ranging from 0.934 (REM) to 0.993 (Wake). However, these improvements came with increased memory usage, reaching 573.05 MB at the final stage of result assembly and confusion matrix generation. This makes the complete feature configuration better suited for performance-critical or offline applications in environments where computational resources are abundant.

Regarding the sleep staging results, the high accuracy in identifying the N3 sleep stage reflects the strong physiological distinctiveness of slow-wave activity in this stage. In contrast, the comparatively lower precision for REM classification can be interpreted in light of REM’s overlapping spectral and temporal characteristics with both Wake and N1, a well-known challenge in clinical sleep scoring [46]. These findings suggest that while the model robustly captures physiologically distinct stages, it inherits the same ambiguities encountered by human scorers in borderline conditions, reinforcing both the reliability and the clinical realism of the proposed approach.

When compared with earlier studies that utilized both frontal and occipital channels, the proposed deep learning model demonstrated superior performance using significantly less input—only one frontal channel and either a reduced or complete feature set. A study with a hybrid model that combines Ensemble Empirical Mode Decomposition (EEMD) with XGBoost, applied to the Sleep-EDF, DREAMS, and SHHS datasets. Using derivations such as Pz-Oz, Cz-A1, and C4-A1, they achieved accuracies exceeding 83% across all datasets, with results including 91.9% on the Sleep-EDF (5-class) dataset [55]. Another study further improved performance by using the Null Space Pursuit (NSP) decomposition algorithm on a single-channel EEG (Pz-Oz), reporting impressive accuracies of 93.59% (4-class) and 92.98% (5-class) on the Sleep-EDF dataset, as well as similarly high values on the DREAMS and SHHS datasets [63,64]. The proposed deep learning model demonstrated superior performance for the 5-class with overall accuracies of 93.5% and 94.7% using the reduced feature sets (94% and 98% cumulative explained variance, respectively) and 97.9% using the complete feature set, using significantly less input—only one frontal channel and either a reduced or complete feature set. Results highlight the effectiveness of the two-step modeling approach in deriving meaningful insights from compact EEG data while enabling accurate classification with low system complexity.

Although limiting the analysis to frontal EEG signals omits certain informative components (e.g., occipital alpha rhythms), the model nonetheless achieved state-of-the-art performance in five-class sleep staging. The combination of LSTM-based temporal modeling with effective feature reduction proves especially advantageous for developing cost-effective, portable, and scalable solutions for home-based sleep monitoring. In further research, we will thoroughly evaluate multi-head attention mechanisms and explore additional regularization strategies to investigate their potential for further enhancing the model’s performance. Future work will focus on validation using other public datasets (such as Sleep-EDF, MASS, and ISRUC-Sleep), evaluating performance across diverse sleep datasets, and reporting inter-rater agreement and validation results using Cohen’s kappa. Additionally, future development will include deploying the model on wearable hardware platforms to enable real-time sleep analysis.

## 6. Conclusions

This work presents an integrated approach for EEG-based sleep monitoring that combines experimental signal acquisition with a deep learning-based sleep staging. The proposed experimental setup, based on bipolar frontal derivations (Fp2-Fp1, F8-F7, and F7-Fpz) and a 3D-printed earbud equipped with textile electrodes (Ein–Eout derivation), enabled the effective acquisition of EEG signals and the extraction of relevant features for sleep staging and the detection of sleep disorders. A comprehensive set of features, drawn from the literature and spanning time, frequency, and non-linear domains, was selected and validated using the open-source dataset comprising approximately 56 h of annotated EEG recordings from 7 individuals. Feature selection using the mRMR algorithm (with a relevance score threshold of 0.01), followed by PCA retaining 98% of the cumulative variance, reduced the feature set from 100 to 13 elements, which were then used in a two-step LSTM-based classification framework. To assess the impact of further dimensionality reduction on model performance, a 94% cumulative explained variance threshold was applied, resulting in a reduced feature set of 11 PCs. Experimental results with overall accuracies of 93.6% and 94.7% using the reduced feature sets (94% and 98% cumulative explained variance thresholds, respectively) and 97.9% using the complete feature set, confirm the discriminative power of the selected features and the excellent performance of the proposed two-stage deep learning algorithm for sleep staging. These results provide strong validation of the methodology, establishing a solid foundation for translating the approach from controlled experimental settings to practical applications. These findings support the use of EEG systems in real-world applications, offering a promising alternative to traditional polysomnography.

## Figures and Tables

**Table 1:** Comparison of scientific works analyzed in Section 2.1 regarding systems for acquiring/processing the forehead EEG signal.

Reference	Electrode Position	Material of the Electrode	Number of Channels	Device Objective	Battery Life
S. Matsumori et al. [16]	2–7 channels from equally spaced forehead electrodes	Ag	3	Sleep staging	12 h
J.A. Onton et al. [17]	Fp1-AFz, Fp2-Fp1, and Fp2-AFz	Hydrogel	3	Sleep staging	14 h
P.J. Arnal et al. [18]	O1, O2, FpZ, F7, and F8	Ag/AgCl	5	Sleep staging and quality	25 h
M.R. Carnerio et al. [19]	AF8, AF10, FP10, FP2, FP1, FP9, AF7, AF9	Conductive stretchable ink	24	EEG acquisition	24 h
Z. Wang et al. [20]	F7, F8, T3, T4, O1 and O2	Flexible, claw-shaped dry electrodes	6	Sleep monitoring	^a^ N. A.
H. Guo et al. [21]	Fh1, Fh2	Dry electrodes	3	Sleep staging	^a^ N. A.
A. Leino et al. [22]	Fp1/Fp2	Ag/AgCl	1	Sleep staging	^a^ N. A.

^a^ N. A.: not available.

**Table 2:** Comparison of analyzed scientific works regarding systems for acquiring/processing ear/in-ear EEG.

Reference	Electrode Position	Type of Electrodes	Number of Channels	Device Objective	Algorithm
G. Palo et al. [23]	In-ear	Dryode ink electrodes	1	To compare the in-ear EEG with standard PSG for sleep staging	JSD-FSI
D. Looney et al. [26]	In-ear (diametrically opposite)	Flexible conductive fabric	2	Sleep staging	AASM (The American Academy of Sleep Medicine) sleep-scoring
S. Mandekar et al. [27]	Out-ear (spaced 120° apart)	Flexible conductive fabric	3	EEG acquisition	Alpha band power correlation
YR. Tabar et al. [29]	In-ear	Titanium, IrO_2_	2	Sleep monitoring	RF Classifier
A. Nguyen et al. [30]	In-ear	Conductive silver leaves, adhesive gel, and fabric	1	Sleep staging	NMF

**Table 3:** Explained and cumulative explained variance of the principal components. A threshold of 94% was applied to the cumulative explained variance of the PCs. The retained PCs are highlighted in green, while the discarded ones are highlighted in orange.

Principal Component	Explained Variance [%]	Cumulative Variance [%]
PC1	16.9	16.9
PC2	13.2	30.1
PC3	12.2	42.3
PC4	10.8	53.1
PC5	8.7	61.8
PC6	7.5	69.3
PC7	6.6	75.9
PC8	6.0	81.8
PC9	4.9	86.7
PC10	4.3	91.1
PC11	3.8	94.9
PC12	2.9	97.8
PC13	2.2	100.0

**Table 4:** Features used for sleep staging, sleep deprivation assessment, and psychological stress analysis from EEG signals.

Analysis Objective	Features
Sleep staging [13,14,26,29,30,37,44,45,55,56,57,58,59]	Maximum Value, Minimum Value, Mean Value, Median, Root Mean Square, 25th, 50th, 75th Percentile, Variance, Skewness, Kurtosis [55], Hjorth Parameters [56], ZCR [26], AAC, Clearance Factor [29], Interquartile Range [30], SSI [37], Total Power [57], Power Ratios [13], Dominant Frequency [44], Slow Wave Indexes [45,59], Harmonic Parameters [14], Band Energy [13], Spectral Slope [58]
Sleep deprivation and disorders [13,15,26,33]	ZCR [26], Total Power [12,33], Band Energy [15]
Psychological stress due to sleep deprivation [13,56,57,60]	Hjorth Parameters [56], RSP [43], Spectral Entropy, LZ Complexity, Rényi Entropy, SVD Entropy [13,57,60]

**Table 5:** Absolute power values of four EEG derivations in the three conditions.

	Open Eyes	Closed Eyes	Evoked1
Fp2-Fp1	2.60 × 10^−10^ W	6.89 × 10^−11^ W	4.69 × 10^−11^ W
F8-F7	5.39 × 10^−9^ W	3.39 × 10^−10^ W	5.91 × 10^−10^ W
F7-Fpz	1.59 × 10^−9^ W	3.24 × 10^−10^ W	2.90 × 10^−10^ W
Ein-Eout	7.41 × 10^−9^ W	5.58 × 10^−9^ W	4.37 × 10^−9^ W

**Table 6:** Classification report for 5-stage sleep classification using the two-step LSTM classifier using the feature set with 13 features (98% cumulative explained variance).

	Precision	Recall	F1-Score	Support for Each Class
Wake	0.979	0.974	0.977	6752
REM	0.747	0.991	0.852	1478
N1	0.993	0.903	0.946	2039
N2	0.967	0.902	0.934	2699
N3	0.992	0.917	0.953	1175
Macro avg	0.936	0.938	0.932	14,143 (total support)
Weighted avg	0.955	0.947	0.949	14,143 (total support)
Accuracy	**0.947 (i.e., 94.7%)**			14,143 (total support)

**Table 7:** Classification report for 5-stage sleep classification using the two-step LSTM classifier using the reduced feature set with 11 features (94% cumulative explained variance).

	Precision	Recall	F1-Score	Support for Each Class
Wake	0.977	0.969	0.973	6752
REM	0.711	0.992	0.828	1478
N1	0.958	0.914	0.936	2039
N2	0.983	0.914	0.936	2699
N3	0.964	0.899	0.930	1175
Macro avg	0.919	0.925	0.916	14,143 (total support)
Weighted avg	0.946	0.936	0.938	14,143 (total support)
Accuracy	**0.936 (i.e., 93.6%)**			14,143 (total support)

**Table 8:** Classification report for 5-stage sleep classification using the two-step LSTM classifier (complete feature set).

	Precision	Recall	F1-score	Support for each class
Wake	0.992	0.993	0.993	6816
REM	0.882	0.991	0.934	1484
N1	0.990	0.957	0.974	2039
N2	0.992	0.967	0.979	2699
N3	0.993	0.940	0.966	1175
Macro avg	0.970	0.970	0.969	14,213 (total support)
Weighted avg	0.980	0.979	0.979	14,213 (total support)
Accuracy	**0.979 (i.e., 97.9%)**			14,213 (total support)

**Table 9:** Mean values of some features for EEG signals acquired from the Fp2-Fp1 derivation in the four conditions: “open eyes”, “closed eyes”, “evoked1”, and “evoked2”.

Feature	Open Eyes	Closed Eyes	Evoked1	Evoked2
Variance [V^2^]	5.6126 × 10^−10^	2.7623 × 10^−10^	9.2886 × 10^−11^	1.3600 × 10^−9^
Hjorth activity [V]	5.6126 × 10^−10^	2.7623 × 10^−10^	9.2886 × 10^−11^	1.3600 × 10^−9^
Hjorth mobility [Hz]	353.03	366.35	570.07	563.47
Hjorth complexity	4.7210	4.9504	2.7321	2.6554
RSP in alpha band	8.4320 × 10^−3^	4.0915 × 10^−2^	6.2681 × 10^−2^	3.3350 × 10^−2^
DSI	220.19	6.9247	2.6467	11.752
TSI	1.1033 × 10^−2^	0.1213	0.2635	9.6001 × 10^−2^
ASI	1.1308 × 10^−2^	5.2976 × 10^−2^	0.1166	5.7637 × 10^−2^
Spectral slope in delta band [V/Hz]	−6.0904	−3.0129	−2.2365	−3.2671
Spectral slope in theta band [V/Hz]	−1.4609	−2.6125	−1.2880	−1.2904
Spectral slope in alpha band [V/Hz]	−0.6966	−0.6409	−1.0234	−0.5779
Spectral slope in beta band [V/Hz]	1.0776	−6.6134 × 10^−2^	−2.3219 × 10^−2^	−0.2998
Spectral slope in gamma band [V/Hz]	−0.36971	−0.4077	−0.6696	1.0208
Delta–theta ratio	392.23	11.283	4.5284	19.349
Delta–alpha ratio	555.32	24.840	8.3635	33.929
Delta–beta ratio	130.06	11.605	2.3912	7.3553
Delta–gamma ratio	126.81	16.769	3.2509	7.3014
Theta–delta ratio	1.1569 × 10^−2^	0.1295	0.3051	0.1052
Theta–alpha ratio	1.6319	2.9363	2.4523	2.0191
Theta–beta ratio	0.3858	1.3408	0.6628	0.5853
Theta–gamma ratio	0.4040	1.8459	0.8758	0.5804
Spectral Entropy	8.8508 × 10^−2^	0.1904	0.4576	0.3377
Renyi Entropy	2.6029	1.5355	1.2446	2.2815
SVD Entropy	0.1610	0.4183	0.7065	0.6595

**Table 10:** Absolute Coefficient of Variation (CV) statistics across sleep stages.

Sleep Stage	Median CV (%)	Mean CV (%)	Standard Deviation
Wake	125.05	1371.63	6967.04
N2	101.07	1259.30	4897.27
N1	93.11	420.19	1378.86
REM	87.20	5116.69	28,199.65
N3	71.56	1041.35	9146.77

## Data Availability

The data are available upon request.

## Supplementary Materials

The following supporting information can be downloaded at: https://www.mdpi.com/article/10.3390/s25196021/s1, Figure S1: Figure 1a_uncropped; Figure S2: Figure 1b_uncropped; Figure S3: Figure 1c_uncropped; Figure S4: Figure 2a_uncropped; Figure S5: Figure 2b_uncropped.

## Abbreviations

The following abbreviations are used in this manuscript:

EEG	Electroencephalography
mRMR	Minimum Redundancy Maximum Relevance
PCA	Principal Component Analysis
LSTM	Long Short-Term Memory
BOAS	Bitbrain Open Access Sleep
PSG	Polysomnography
ECG	Electrocardiogram
EOG	Electrooculogram
EMG	Electromyogram
SpO_2_	Blood Oxygen Saturation
PPG	Photoplethysmography
ML	Machine Learning
DL	Deep Learning
VEP	Visual Evoked Potentials
LS	Light Sleep
DS	Deep Sleep
RF	Random Forest
REM	Rapid Eye Movement
NREM	Non-Rapid Eye Movement
JSD-FSI	Jensen-Shannon Divergence Feature-based Similarity Index
AASM	The American Academy of Sleep Medicine
LIBS	Lightweight In-ear Biosignal Sensing System
NMF	Negative Matrix Factorization
ADCs	Analog-To-Digital Converters
FFT	Fast Fourier Transform
PRVEP	Pattern Reverse Visual Evoked Potential
RMS	Root Mean Square
ZCR	Zero-Crossing Rate
AAC	Average Amplitude Change
IQR	Interquartile Range
SSI	Simple Square Integral
RSP	Relative Spectral Power
SWI	Slow Wave Index
ASI	Alpha Slow Wave Index
SEFd	Spectral Edge Frequency difference
AP	Absolute Power
SVD	Singular Value Decomposition
LZC	Lempel–Ziv Complexity
MI	Mutual Information
PSD	Power Spectral Density
DAR	Delta–alpha ratio
DTR	Delta–theta ratio
DTABR	Delta–theta–alpha–beta ratio
PCs	Principal Components
DSI	Delta Slow Wave Index
TSI	Theta Slow Wave Index
CV	Coefficient of Variation
