# Supplementary figures and images for: Forehead and In-Ear EEG Acquisition and Processing: Biomarker Analysis and Memory-Efficient Deep Learning Algorithm for Sleep Staging with Optimized Feature Dimensionality
Source: Sensors (Basel). 2025 Oct 1;25(19):6021. doi: 10.3390/s25196021 (PMC12526609; doi:10.3390/s25196021)

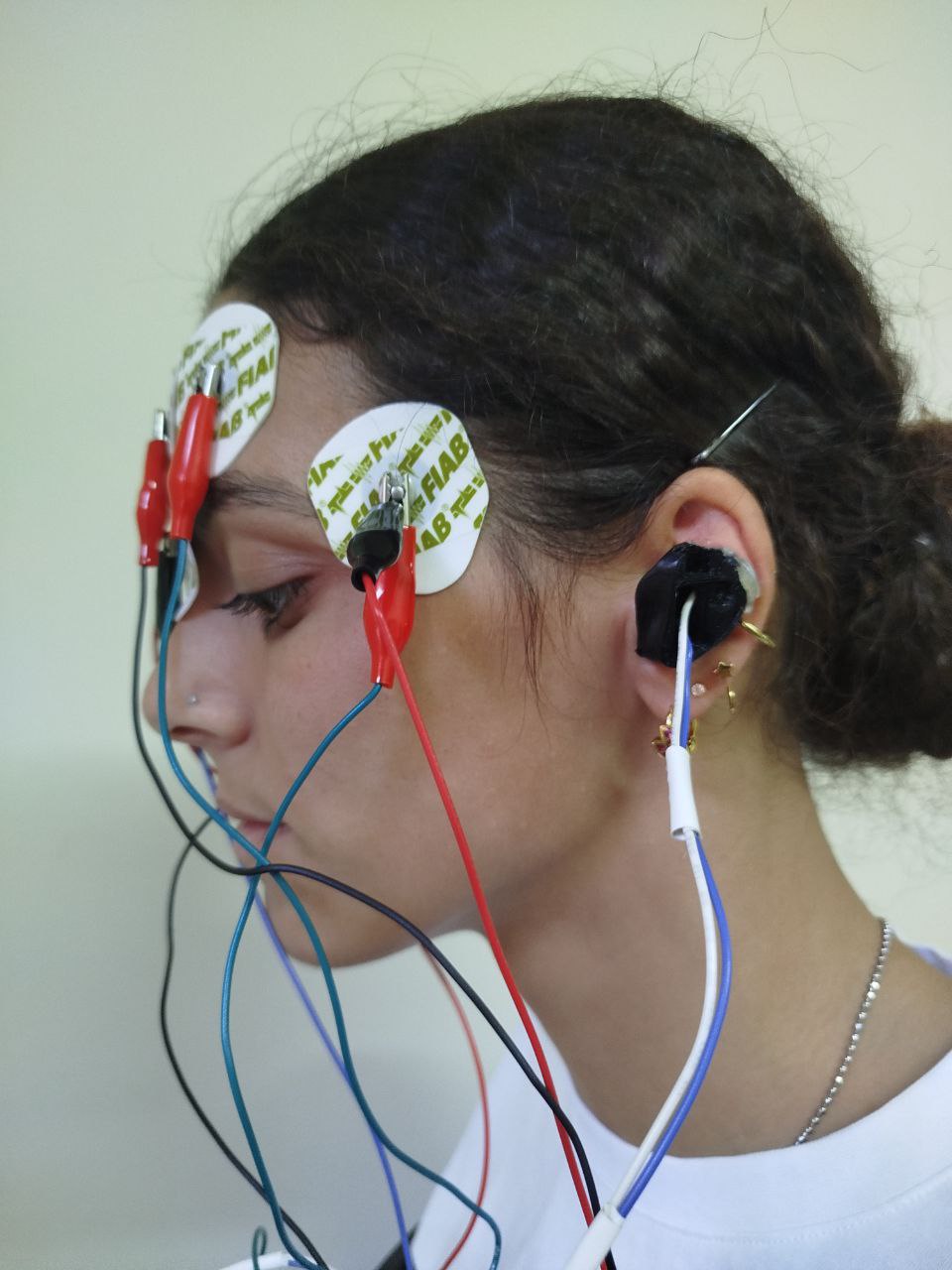

Supplement: Supplementary file 1 [file sensors-25-06021-s001.zip › Figure S1- Figure 1a_uncropped.jpg]

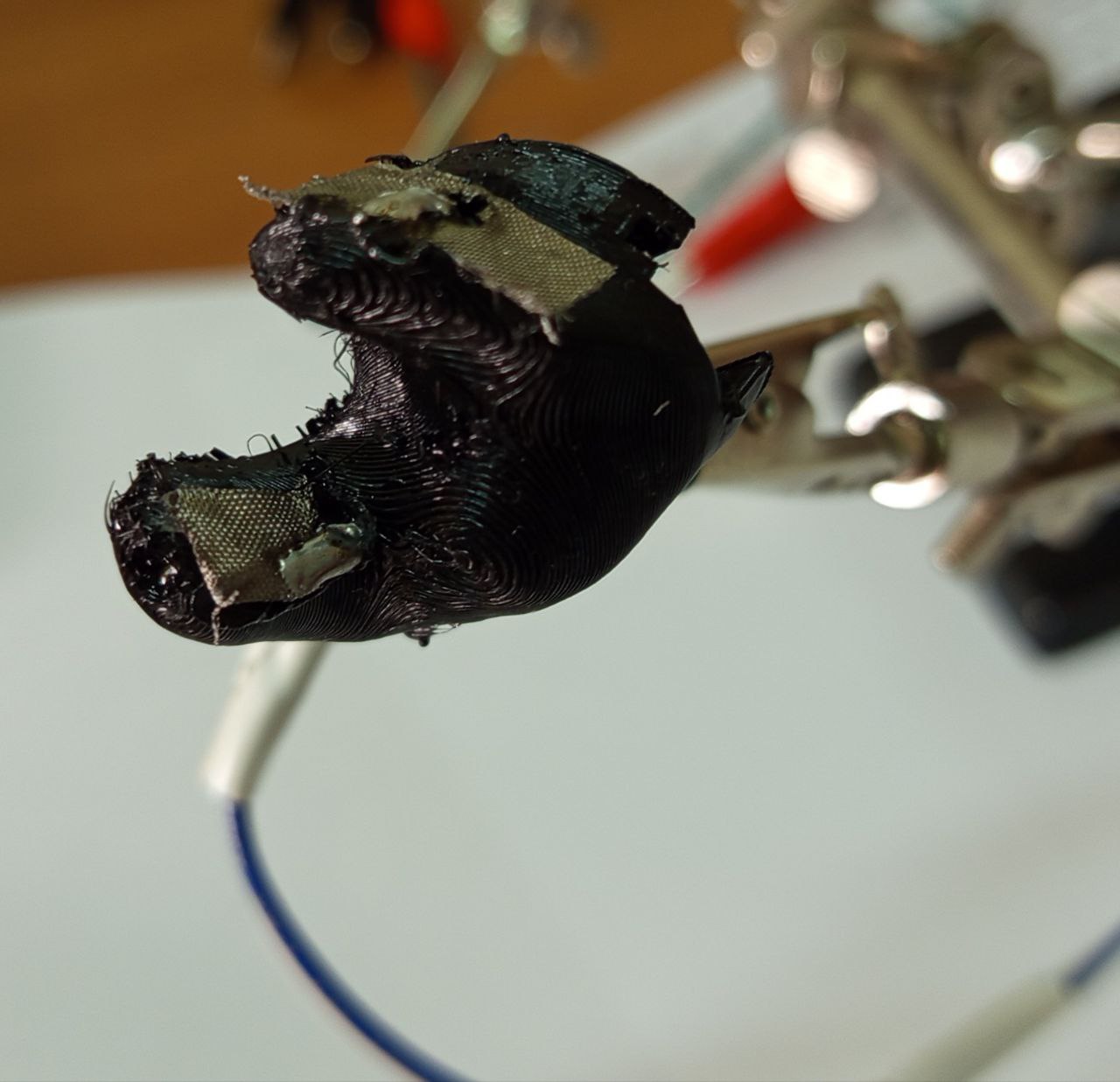

Supplement: Supplementary file 1 [file sensors-25-06021-s001.zip › Figure S2 - Figure 1b_uncropped.jpg]

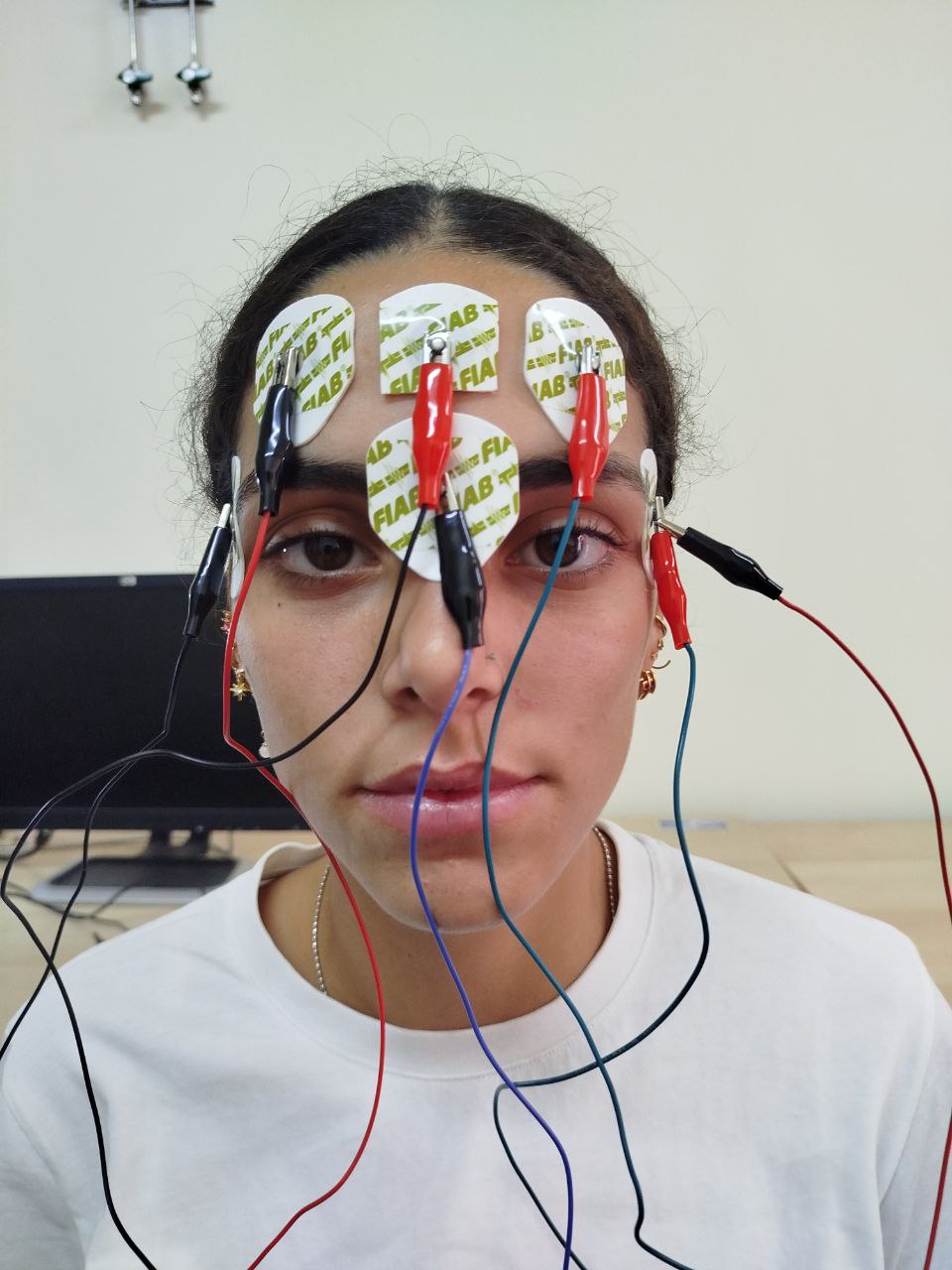

Supplement: Supplementary file 1 [file sensors-25-06021-s001.zip › Figure S3 - Figure 1c_uncropped.jpg]

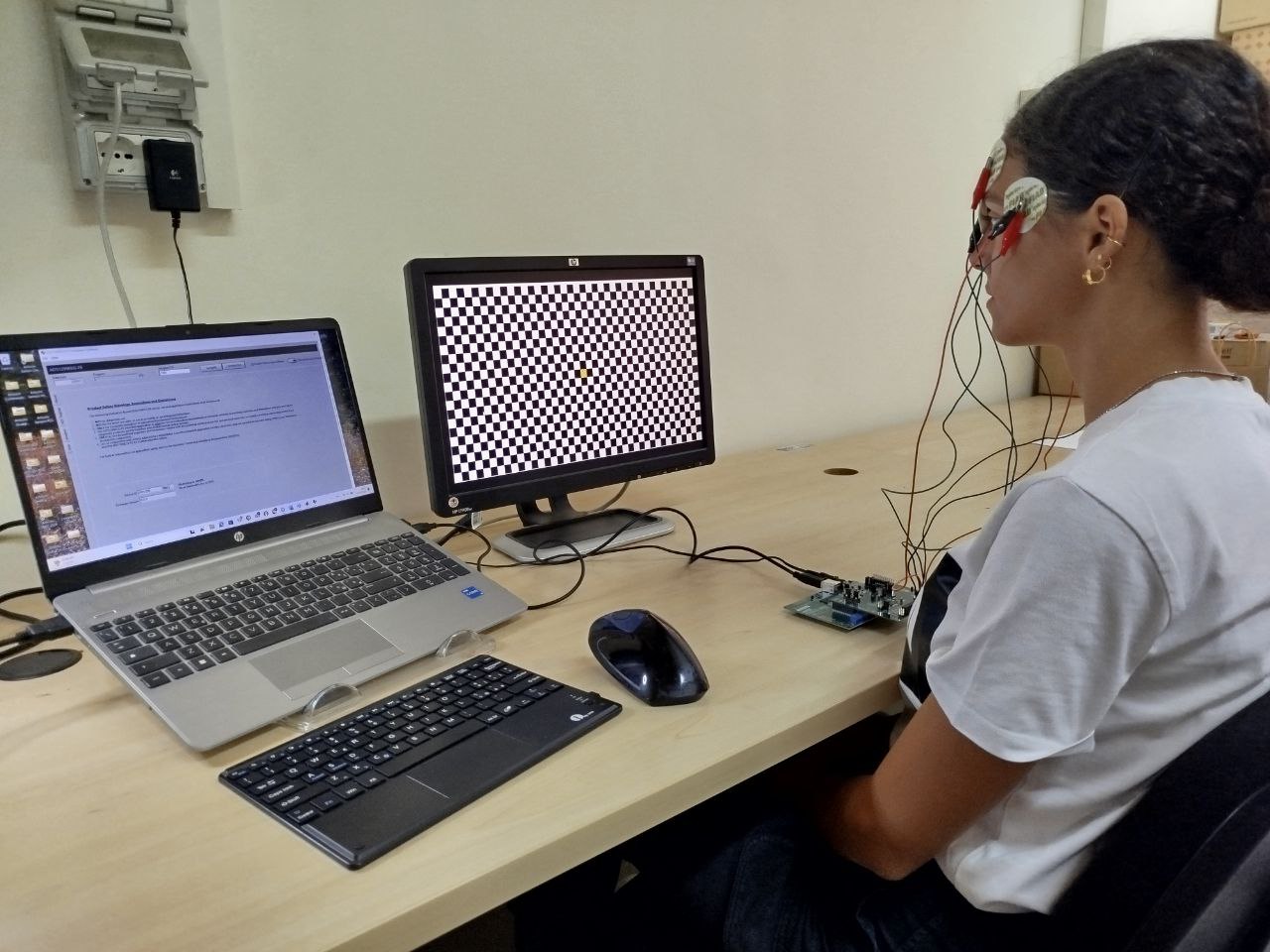

Supplement: Supplementary file 1 [file sensors-25-06021-s001.zip › Figure S4 - Figure 2a_uncropped.jpg]

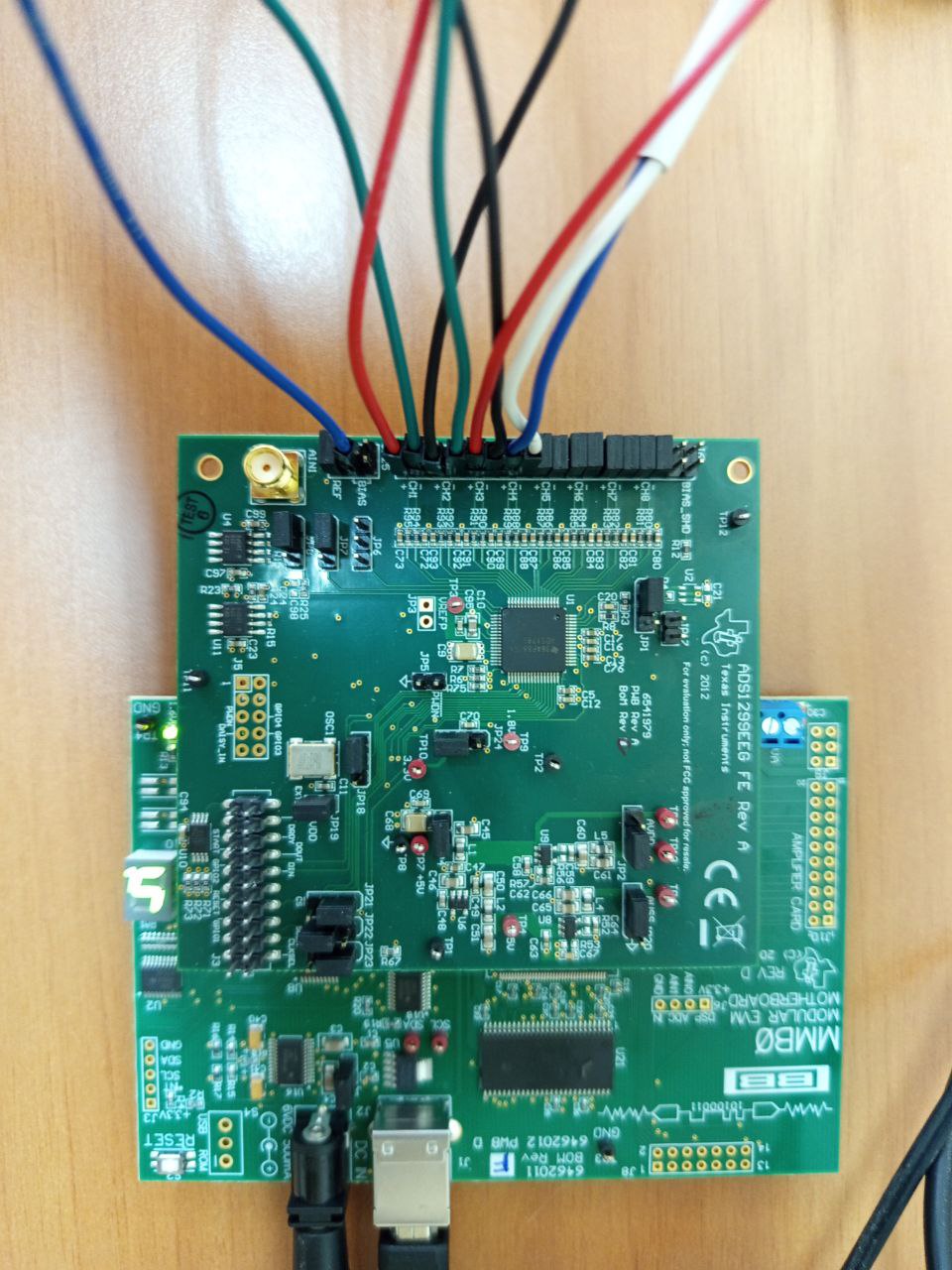

Supplement: Supplementary file 1 [file sensors-25-06021-s001.zip › Figure S5 - Figure 2b_uncropped.jpg]
